# Direct and indirect effects of urban gardening on aboveground and belowground diversity influencing soil multifunctionality

**DOI:** 10.1038/s41598-019-46024-y

**Published:** 2019-07-05

**Authors:** Simon Tresch, David Frey, Renée-Claire Le Bayon, Paul Mäder, Bernhard Stehle, Andreas Fliessbach, Marco Moretti

**Affiliations:** 1 Research Institute of Organic Agriculture (FiBL), Department of Soil Sciences, Ackerstrasse 113, 5070 Frick, CH Switzerland; 2 Swiss Federal Research Institute WSL, Biodiversity and Conservation Biology, Zürcherstrasse 111, 8903 Birmensdorf, CH Switzerland; 3 University of Neuchâtel, Institute of Biology, Functional Ecology Laboratory, Rue Emile-Argand 11, 2000 Neuchâtel, CH Switzerland; 4 ETHZ, Department of Environmental System Science, Institute of Terrestrial Ecosystems, Universitaetstrasse 16, 8092 Zurich, CH Switzerland; 5 University of Konstanz, Department of Biology, Ecology, Universitätstrasse 10, 78464 Konstanz, DE Germany

**Keywords:** Urban ecology, Ecosystem services

## Abstract

Urban gardens are popular green spaces that have the potential to provide essential ecosystem services, support human well-being, and at the same time foster biodiversity in cities. We investigated the impact of gardening activities on five soil functions and the relationship between plant (600 spp.) and soil fauna (earthworms: 18 spp., springtails: 39 spp.) in 85 urban gardens (170 sites) across the city of Zurich (Switzerland). Our results suggest that high plant diversity in gardens had a positive effect on soil fauna and soil multifunctionality, and that garden management intensity decreased plant diversity. Indices of biological activity in soil, such as organic and microbial carbon and bacterial abundance, showed a direct positive effect on soil multifunctionality. Soil moisture and disturbance, driven by watering and tilling, were the driving forces structuring plant and soil fauna communities. Plant indicator values proved useful to assess soil fauna community structure, even in anthropogenic plant assemblages. We conclude that to enhance soil functions, gardeners should increase plant diversity, and lower management intensity. Soil protective management practices, such as applying compost, mulch or avoiding soil tilling, should be included in urban green space planning to improve urban biodiversity and nature’s contribution to people.

## Introduction

Maintaining functional and biodiverse urban green spaces is fundamental for liveable cities (cf. SDG 11^1^). Urban gardens are a major component of urban green spaces in many countries^2,3^. They are heterogeneous in structure, but despite their relatively small size they provide critical habitat resources and increase the connectivity of urban landscapes^4^. Garden management creates diverse garden land-use types including perennially vegetated habitats such as lawns or annually vegetated habitats such as vegetable beds^2^. These diverse microhabitats support urban biodiversity and have the ability to provide nature’s contributions to people^5,6^. The worldwide increase in human population is expected to take place mainly in urban areas^7^, while growing cities often expand onto fertile agricultural soils, thus challenging the supply of fresh food in the future^8^. There is a great potential for producing food in urban gardens and at the same time to provide other ecosystem services (ES) in densely populated cities^9^. It is estimated that urban farming delivers food for approximately 800 million people^10^, although the current global scale is difficult to assess^11^. However, hundreds of millions of citizens rely on urban agriculture for part of their nourishment^12^. Nonetheless, urban garden soils are also important for regulating soil functions such as water storage (flood control^13^), C and N storage^14^, pollination^15^, soil formation^16^, pest control^17^, or to decrease urban heat island intensity^18^ and provide habitats for many species even in densely urbanised areas^6^. From a sociological perspective, urban gardens are important for recreation, well-being, and social interaction^19^.

Urban gardening has a long tradition in many countries around the world^20^. As a consequence of decades of beneficial soil management practices, such as the application of compost^21^, urban garden soils may not always be as poor in quality and potentially polluted as other urban soils^3,22^. Despite the importance of gardens for urban biodiversity^6^, information on the ecological importance of allotment and domestic gardens is still scarce compared to public green spaces^2,23^. However, there is a large body of evidence that biodiversity drives ecosystem processes and related services in aboveground communities^24^, but the functioning of belowground biodiversity is much less understood^25^. Although it has been shown that soil biodiversity is linked in multiple ways with aboveground biodiversity^26,27^, further investigation is needed to better understand these relationships. Garden soils are strongly influenced by human activities^3,22,28^, but they are also affected by the past land-use, the degree of disturbance or climate related drivers such as the urban heat island effect^29^. Soil functions are provided and controlled by a large variety of soil organisms^30^, also in urban soils^28^, where the frequency of soil disturbance is often high^29^. Changes in community composition of soil fauna in both alpha and beta diversity^31^, for instance due to soil disturbance, can impair soil functions such as organic matter decomposition or nutrient retention^32^. The interactions of aboveground and belowground species, driving ecosystem functions, at least at the local scale^33^, are mainly linked via plants^34^. However, still very little is known about this relationship between aboveground and belowground diversity and associated soil functions^35^, especially for garden soils^36^.

The ability of an ecosystem to provide multiple functions, so-called multifunctionality^24^, can be calculated as indices based on the functions of interest^37^. Such measures of multifunctionality (i.e. the averaging approach), have been used to analyse a wide range of ecosystem drivers^38^, such as soil characteristics^39^, habitat diversity^40^, climate^41^, or management practices in agriculture^42^ and even in constructed ecosystems such as green roofs^43^. Here, we focus on five independent measurements for calculating soil multifunctionality ranging from aboveground^44^ and belowground^27^ litter decomposition, to nutrient supply for plant growth^45^ and water regulation, such as water storage capability^46^.

Research on urban garden soils has recently received increased attention^3,17,28,47,48^, especially with regard to human health and well-being^29^. However, our understanding of the complex interactions between management practices, soil biodiversity and soil functioning is still scarce^49^. In this study, we focus on gardening activities in the two most dominant garden types of Zurich (CH), allotment and domestic gardens, and assess the interactions between aboveground diversity of plants and belowground diversity of soil fauna. We investigated earthworms (Oligochaeta: Lumbricidae), representing soil macrofauna species and springtails (Hexapoda: Collembola), representing soil mesofauna species, as indicators for soil functioning^50^ and assessed the impacts of urban gardening on soil multifunctionality. Earthworms are generally described as ecosystem engineers^50^, due to their impact on soil structure and quality, at least in temperate soils^51^. They are important indicator organisms for soil functions^52^, soil disturbance, and management practices^50^. It has been shown that also in urban ecosystems such as parks or urban gardens, they are sensitive indicators of anthropogenic management intensity^47^. Springtails are a key group of microarthropods^50^ and can be used as indicators of sustainable land-use, soil quality^27^, or the use of pesticides^53^. Moreover, they are used to assess soil functionality^54^ and the impact of environmental factors^55^ on soil biodiversity. In addition, we assessed soil microfauna by biological soil measurements, such as basal respiration, microbial biomass and gene copy numbers of bacteria and fungi (Table 1).

**Table 1:** Soil characteristics describing the soil quality of urban garden sites used as explanatory variables in the SEM.

Variables	Description	PC1	PC2	PC3	PC4
**Physical soil characteristics**					
BD [g cm^−3^]	Soil bulk density	0.39	−0.31	0.06	−0.15
PR [MPa]	Penetration resistance	0.03	0.39	0.03	−0.31
SA [%]	Soil stable aggregates	−0.26	0.44	−0.04	0.15
**Chemical soil characteristics**					
Fe [mg kg^−1^]	Iron content	−0.41	−0.17	0.29	0.07
K [mg kg^−1^]	Potassium content	−0.35	−0.33	0.03	−0.05
Mn [mg kg^−1^]	Manganese content	0.01	0.13	0.41	−0.04
Mg [mg kg^−1^]	Magnesium content	−0.13	−0.24	−0.44	0.18
P [mg kg^−1^]	Phosphorus content	−0.22	−0.34	0.41	0.09
pH	Soil pH	0.18	−0.07	−0.49	−0.04
**Biological soil characteristics**					
C_mic_ [mg kg^−1^]	Microbial biomass carbon	−0.37	0.35	−0.13	−0.07
C_org_ [%]	Soil organic carbon content	−0.39	−0.20	−0.33	0.18
Bacteria [gene copies]	16S bacterial gene copy number	−0.31	0.05	−0.11	−0.60
Fungi [gene copies]	18S fungal gene copy number	−0.03	−0.26	−0.04	−0.64
	Eigenvalue	2.7	2.4	1.7	1.5
	Explained variance [%]	20.8	18.4	13.3	11.7

The first four PCA axes scores (PC1–PC4; Fig. S4) were used (Kaiser-Guttman criteria) as explanatory variables in the SEM (Fig. 2), explaining 64.2% of the total variation.

**Figure 1:**
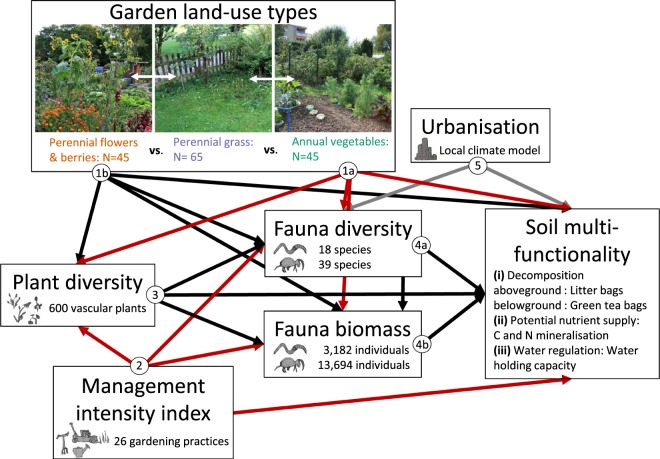
A priori SEM model with hypothesised direct and indirect effects of urban gardening on soil multifunctionality. Expected positive relationships are given in black and negative ones in red, grey arrows represent both positive and negative effects. We expected that *annual vegetables* (arrows 1a) will negatively influence plant and soil fauna as well as soil multifunctionality compared to *perennial grass* sites, while perennial flowers (arrows 1b) will show positive effects. Management intensity (arrows 2) is expected to negatively affect plant diversity and soil fauna as well as soil multifunctionality. Higher plant diversity (arrows 3) is hypothesised to have a positive effect on soil fauna and soil multifunctionality. Soil fauna diversity and biomass (arrows 4a & 4b) are also expected to have a positive effect on soil multifunctionality. Urbanisation (arrows 5) might have a positive or negative effect on soil fauna and soil multifunctionality. Expected effects of soil characteristics (arrows 6) can be found in Fig. S9.

The overall objective of our study was to investigate impacts of garden management practices (management intensity index, garden land-use types) on aboveground plant diversity and belowground diversity of soil fauna, and their direct and indirect effects on soil multifunctionality. We hypothesised (cf. a priori structural equation model (SEM) Fig. 1) that (i) intensive soil management will reduce the diversity of both plant and soil fauna and negatively affect soil multifunctionality (arrows 1 & 2). We assumed that (ii) aboveground and belowground diversity are also linked in urban garden ecosystems and therefore expected that a higher diversity of plants will have a positive effect on both soil fauna and soil multifunctionality (arrows 3). Furthermore, we expected that (iii) soil fauna diversity (arrow 4a) and biomass (arrow 4b) will have a direct positive effect on soil multifunctionality. Additionally, we assumed an influence of (iv) soil characteristics and (v) urbanisation on soil multifunctionality (arrows 5 & 6).

In a second step, we analysed soil fauna community structure. We expected that frequently disturbed soils would have the lowest species diversity within (alpha diversity) and among (beta diversity) garden sites, including a high community evenness and beta diversity mainly driven by species loss (nestedness) rather than species replacement (turnover). For the plant community, we expected highest alpha and beta diversity for garden sites with high planting activities, including a high species turnover component for beta diversity. Furthermore, we investigated impacts of management practices on soil fauna community composition and on soil fauna disturbance indices.

## Results

### Urban gardening effects on soil fauna and soil multifunctionality

**Figure 2:**
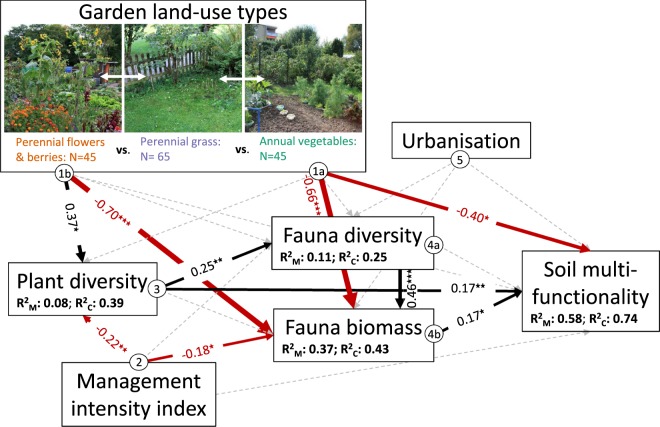
Final most parsimonious SEM connecting garden management (land-use types, management intensity), urbanisation, plant and soil fauna diversity with soil multifunctionality (AICc = 288.3, Fisher’s C = 24.3, P = 0.93). Arrows represent unidirectional relationships among variables. Black arrows denote significantly (p < 0.05) positive and red arrows significantly negative relationships (Table 3). Dashed grey arrows represent non significant relationships (p > 0.05). The thickness of paths has been scaled based on the magnitude of the standardised regression coefficient. Conditional R^2^s, based on the variance of both the fixed and random effects, as well as marginal R^2^s, based on the fixed effect parts for each component models are given in the boxes of the response variables. Soil multifunctionality consists of five measurements related to important soil functions. Soil characteristics are included in Fig. S10.

The SEM based on our a priori expectations (Fig. 1) of urban gardening effects on aboveground and belowground diversity and soil multifunctionality met the criteria of Fisher’s C statistic^56^ (Fisher’s C = 30.7; p = 0.80; AICc = 286.8). The model included one significant missing path^57^ between PC1 and soil fauna diversity (SEM; 0.18; p = 0.03). With the inclusion of this path the overall model fit of the SEM improved (Fisher’s C = 24.3; p = 0.93; AICc = 288.3), with marginal differences in the AICc (1.5). Overall, the strongest relationships in the SEM originated from garden land-use types, influencing plant diversity, fauna biomass and soil characteristics (PC2) and soil multifunctionality both in indirect and direct ways (Figs 2 and S10). The strongest effects on soil multifunctionality came from soil PC1 (SEM; −0.61; p =< 0.001), represented by lower loads of C_mic_, C_org_, bacteria, Fe and K, but higher soil bulk density values (Fig. S4). Soils with increased C_mic_, C_org_, bacteria, Fe and K, but lower bulk density values thus covaried with higher soil multifunctionality. *Annual vegetable* sites showed lower soil multifunctionality values (SEM; −0.40; p = 0.03) compared to *perennial grass* sites. Moreover, we found positive effects of plant diversity (SEM; 0.17; p = 0.01), and fauna biomass (SEM; 0.17; p = 0.02) on soil multifunctionality. Taken together, both significant and non-significant effects explained 74% of the total variation of soil multifunctionality. In addition, we also identified several indirect effects on soil multifunctionality (Fig. 2 and S10, Table S6). We found that plant diversity had a positive indirect effect on soil multifunctionality mediated by increased fauna diversity and fauna biomass. Plant diversity itself was positively affected by *flower & berry* sites (SEM; 0.37; p = 0.04) and negatively by management intensity (SEM; −0.22; p = 0.01), explaining 39% of the variation in plant diversity. A similar pattern was found in high beta diversity values (Table S5) for the plants (0.94 ± 0.001), dominated by a high turnover component (0.92 ± 0.001) and low nestedness component (0.02 ± 0.001), indicating the high variability between garden plots. Moreover, the management intensity indirectly negatively affected soil multifunctionality by decreasing fauna biomass and plant diversity (Fig. 2, Table S6). Plant and soil fauna beta diversity and fauna phylogenetic diversity were not included in the final SEM (cf. Table S10, Fig. S7) due to a large increase in the AICc (588.5) and because it explained only 3% more variance in soil multifunctionality (Fig. S8).

### Urban gardening effects on soil fauna community composition

**Figure 3:**
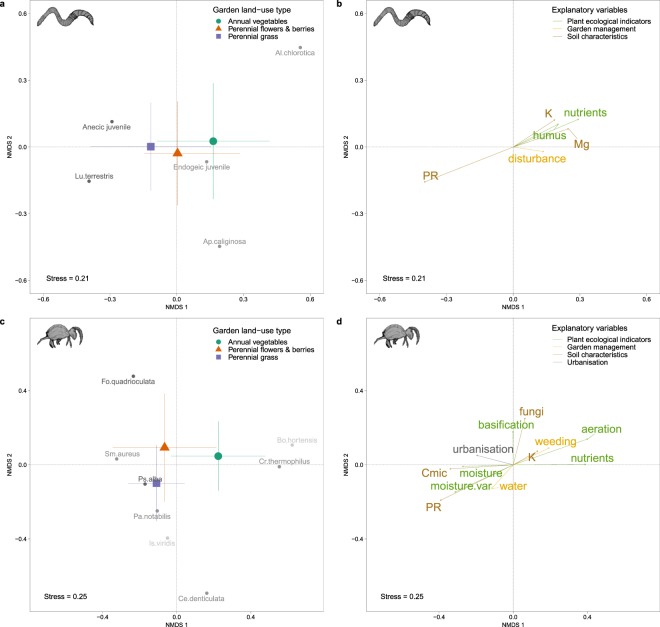
Soil fauna community structure analysis of earthworms (**a**,**b**) and springtails (**c**,**d**). Soil fauna species are coloured in grey, corresponding to three ecological categories (Table S1). Only species with a significant (p < 0.001) effect (Table S4) and only significant explanatory variables (p < 0.05) from the PERMANOVA model (Table 1) were fitted. Garden land-use types include standard deviation bars.

We investigated the effects of management practices, plant ecological indicators (Table 2), soil characteristics (Table 1), garden land-use types, and urbanisation on the community composition of both earthworms and springtails (Table 4). Both soil fauna groups were strongly affected by the plant ecological indicator nutrients (PERMANOVA EW; F = 5.9; p =< 0.001, COL; F = 5.4; p =< 0.001), that represented the requirements of nutrient-rich soils for plants that are predominantly found in *vegetable* sites (Fig. 3). Earthworm communities were further affected by the penetration resistance of the soil (PERMANOVA EW; F = 5.3; p =< 0.001), favouring anecic species such as *L*. *terrestris*. Furthermore, plants indicating humus rich soils (PERMANOVA EW; F = 3.0; p = 0.01) favoured the two most abundant earthworm species (Table S1, Fig. 3) *A*. *chlorotica* and *A*. *caliginosa*. Those soils were further associated with higher contents of Mg (PERMANOVA EW; F = 2.1; p = 0.04) and K (PERMANOVA EW; F = 2.7; p = 0.01), resulting from higher soil disturbance (PERMANOVA EW; F = 2.4; p = 0.02) predominantly in *vegetable* sites. In summary, the NMDS ordination was driven by endogeic species *A*. *chlorotica* (NMDS; R^2^ = 0.30; p =< 0.001) and *A*. *caliginosa* (NMDS; R^2^ = 0.13; p =< 0.001), anecic species *L*. *terrestris* (NMDS; R^2^ = 0.17; p =< 0.001), and both endogeic (NMDS; R^2^ = 0.20; p =< 0.001) and anecic (NMDS; R^2^ = 0.19; p =< 0.001) juveniles. Although the garden land-use type was not a significant factor affecting the earthworm community composition, the most abundant species can be allocated to specific land-use types (Fig. 3, Table S1). In *vegetable* sites we primarily found endogeic species such as *A*. *chlorotica* (64.5%), which is tolerant to disturbances^28^ and a pioneer species^58^, endogeic juveniles (41.9%) and *A*. *caliginosa* (45.1%). *Perennial grass* sites were dominated by anecic species such as *L*. *terrestris* (52.5%) and anecic juveniles (47.6%), probably due to deeper and more compacted soils. As expected, we found the lowest earthworm diversity (D_Simpson_) in *vegetable* sites (1.85 ± 0.2) and the highest in *grass* sites (2.57 ± 0.2). The earthworm diversity was driven by endogeic and anecic species but not by epigeic species (Table 4). Additionally, we found the lowest beta diversity (β_JAC_) for earthworms in *vegetables* and the highest in *grass* sites (Fig. S5, Table S5). However, evenness (E_Simpson_) was not highest in *vegetable* sites but in *grass* sites and *flower & berry* sites, where we also observed higher nestedness components (β_JNE_).

**Table 2:** Garden management practices based on the gardener survey (Table S3) and plant ecological indicator values reflecting the plant environmental requirements^81^.

Variables	Description
**Management practices**	
Disturbance	Frequency of soil disturbance
Fertiliser	Frequency of fertiliser application
Management intensity	Garden management intensity gradient
Pesticides	Frequency of pesticide application
Water	Frequency of water application
Weeding	Frequency of weeding
**Plant ecological indicator values**	
Aeration	Supply of oxygen in the soil (from poor (0) to good (1))
Basification	Soil content of H^+^-ions (from acid (0) to alkaline (1))
Humus	Dark organic matter content (humus) (from little (0) to high (1))
Moisture	Soil moisture during the growing season (from dry (0) to wet (1))
Moisture variability	Alternating soil moisture (from less (0) to often (1) alternating)
Nutrients	Soil nutrient availability (from low (0) to high (1))
Root depth	Depth of soil root penetration (from shallow (0) to deep (1))

Plant ecological indicator values are calculated as community weighted means of plant species found on each sampling plot.

**Table 3:** Final most parsimonious structural equation model (SEM; AICc = 156.3, Fisher’s C = 24.3, P = 0.93) indicating direct and indirect effects on soil multifunctionality from garden land-use types, garden management, plant and soil fauna diversity, soil fauna biomass, soil characteristics and urbanisation.

Response	R^2^_C_	R^2^_M_	Predictor	Estimate ± SE	P
**Soil multifunctionality**	0.74	0.58	Soil PC1	−0.61 ± 0.06	<0.001***
			Plant diversity	0.17 ± 0.06	0.01**
			Fauna biomass	0.17 ± 0.07	0.02*
			Vegetables	−0.40 ± 0.20	0.03*
			Soil PC3	0.12 ± 0.06	0.08
			Urbanisation	0.11 ± 0.07	0.12
			Soil PC2	0.12 ± 0.08	0.15
			Management intensity	0.08 ± 0.06	0.22
			Flowers & berries	−0.08 ± 0.10	0.61
			Soil PC4	−0.03 ± 0.06	0.69
			Fauna diversity	−0.01 ± 0.06	0.83
**Fauna diversity**	0.25	0.11	Plant diversity	0.25 ± 0.09	0.005**
			Soil PC1	−0.18 ± 0.08	0.03*
			Urbanisation	−0.15 ± 0.09	0.09
			Vegetables	−0.26 ± 0.20	0.17
			Management intensity	0.08 ± 0.09	0.37
			Flowers & berries	−0.02 ± 0.20	0.92
**Fauna biomass**	0.43	0.37	Fauna diversity	0.46 ± 0.07	<0.001***
			Flowers & berries	−0.70 ± 0.20	<0.001***
			Vegetables	−0.66 ± 0.20	<0.001***
			Management intensity	−0.18 ± 0.07	0.01*
			Plant diversity	0.09 ± 0.07	0.20
			Urbanisation	0.04 ± 0.07	0.56
**Plant diversity**	0.39	0.08	Management intensity	−0.22 ± 0.08	0.01**
			Flowers & berries	0.37 ± 0.20	0.04*
			Vegetables	0.29 ± 0.20	0.09
**Soil PC1**	0.48	0.02	Urbanisation	−0.12 ± 0.10	0.24
			Vegetables	0.17 ± 0.20	0.30
			Management intensity	−0.05 ± 0.08	0.55
			Flowers & berries	0.04 ± 0.20	0.81
**Soil PC2**	0.60	0.44	Vegetables	−1.60 ± 0.10	<0.001***
			Flowers & berries	−0.79 ± 0.10	<0.001***
			Management intensity	0.05 ± 0.06	0.47
			Urbanisation	−0.04 ± 0.07	0.61
**Soil PC3**	0.76	0.01	Vegetables	0.16 ± 0.10	0.16
			Management intensity	0.07 ± 0.07	0.32
			Flowers & berries	−0.02 ± 0.10	0.86
			Urbanisation	−0.01 ± 0.10	0.90
**Soil PC4**	0.47	0.02	Vegetables	0.22 ± 0.20	0.18
			Urbanisation	−0.09 ± 0.10	0.40
			Flowers & berries	−0.04 ± 0.20	0.83
			Management intensity	−0.01 ± 0.08	0.93

R^2^_M_ is based on fixed effects and R^2^_C_ on fixed and random (garden ID) effects. Total estimates of indirect pathways are given in Table S6.

**Table 4:** PERMANOVA of earthworms (PERMANOVA EW; left) and springtails (PERMANOVA COL; right) and management practices, plant ecological indicators, soil characteristics and garden characteristics as explanatory variables.

	Df	Earthworms			Springtails		
		F	R^2^	P	F	R^2^	P
**Management practices**							
Management Intensity	1	1.1	0.01	0.37	1.3	0.01	0.18
Water	1	1	0.01	0.43	2.5	0.01	<0.001**
Fertiliser	1	1.8	0.01	0.09	1.4	0.01	0.14
Pesticides	1	0.6	0.01	0.75	1.3	0.01	0.19
Disturbance	1	2.4	0.01	0.02*	1.2	0.01	0.24
Weeding	1	1.6	0.01	0.11	1.8	0.01	0.04*
**Plant ecological indicators**							
Moisture	1	1.5	0.01	0.15	3.7	0.02	<0.001***
Moisture Variability	1	1.9	0.01	0.06	3.7	0.02	<0.001***
Basification	1	1.4	0.01	0.18	2	0.01	0.02*
Nutrients	1	5.9	0.04	<0.001***	5.4	0.03	<0.001***
Humus	1	3	0.02	0.01**	0.6	0.01	0.82
Aeration	1	1.2	0.01	0.29	1.8	0.01	0.05*
Root depth	1	0.6	0.01	0.82	0.7	0.01	0.74
**Soil characteristics**							
**Physical measurements**							
SA	1	1.9	0.01	0.06	1.8	0.01	0.05
PR	1	5.3	0.03	<0.001***	2.3	0.01	0.01**
BD	1	1.1	0.01	0.36	0.7	0.01	0.81
**Chemical measurements**							
Mg	1	2.1	0.01	0.04*	1.3	0.01	0.2
P	1	1.1	0.01	0.32	0.6	0.01	0.81
Fe	1	1.4	0.01	0.17	0.7	0.01	0.81
K	1	2.7	0.02	0.01*	2.5	0.01	<0.001**
pH	1	0.8	0.01	0.55	1.1	0.01	0.36
Mn	1	0.7	0.01	0.72	0.7	0.01	0.79
**Biological measurements**							
C_org_	1	0.8	0.01	0.6	0.9	0.01	0.52
C_mic_	1	0.6	0.01	0.73	2.1	0.01	0.02*
Fungi	1	0.8	0.01	0.62	1.8	0.01	0.04*
Bacteria	1	0.7	0.01	0.71	0.8	0.01	0.63
**Garden characteristics**							
Land-use type	2	1	0.01	0.41	2.8	0.03	<0.001***
Urbanisation	1	1.4	0.01	0.17	1.8	0.01	0.04*
Residuals	119		0.72			0.69	

SA: Soil stable aggregates, BD: Soil bulk density, PR: Penetration resistance.

Springtails were affected by the plant ecological indicators moisture (PERMANOVA COL; F = 3.7; p =< 0.001) and moisture variability (PERMANOVA COL; F = 3.7; p =< 0.001). These indicate moist soil or alternating soil moisture conditions, favouring species that were more abundant in *grass* sites such as *S*. *aureus* (81.1%) or *P*. *alba* (67.3%) on soils with high penetration resistance (PERMANOVA COL; F = 2.3; p = 0.01) and C_mic_ (PERMANOVA COL; F = 2.1; p = 0.02). All three life forms of springtails were present in the species that had the biggest effect on the community composition (Table S4). *B*. *hortensis* showed the largest effect (NMDS; R^2^ = 0.29; p =< 0.001), and was most often found in *vegetable* sites (75.5%), where also *C*. *thermophilus* (NMDS; R^2^ = 0.13; p =< 0.001) was often present (56.7%), correlating with potassium loads (PERMANOVA COL; F = 2.5; p =< 0.001). Other species driving the community composition of springtails were mostly found in *grass* sites, such as *P*. *notabilis* (85.4%), representing the most abundant (22.9%) springtail species (13,435 individuals) in this survey. Moreover, we found eight springtails (marked with stars in Table S1) which were not included yet in the Fauna Europaea species list, with two new records for Switzerland (*I*. *balteatus* and *I*. *graminis*) according to the available literature and expert opinion (c.f. Table S1). The separation of *flower & berry* sites in the NMDS was mainly driven by *C*. *denticulata* (NMDS; R^2^ = 0.08; p =< 0.001). Moreover, we found a clear effect of garden land-use type (PERMANOVA COL; F = 2.8; p =< 0.001), but also two significant effects of specific garden management practices: applying water (PERMANOVA COL; F = 2.5; p =< 0.001) and weeding (PERMANOVA COL; F = 1.8; p = 0.04). Weeding was more attributed to *vegetable* sites and applying water to *grass* sites in the NMDS ordination, whereas *flower & berry* sites were associated with a higher degree of urbanisation (PERMANOVA COL; F = 1.8; p = 0.04) and with more alkaline soils (basification; PERMANOVA COL; F = 2.0; p = 0.02). As expected, we found lower mean values for the springtail diversity (D_Simpson_, Table S2) in *vegetable* sites (3.3 ± 0.2) compared to *grass* sites (3.8 ± 0.1). Beta diversity (β_JAC_) was highest for *flower & berry* sites with a high turnover in comparison to the nestedness component (Fig. S5, Table S5). Springtail evenness (E_Simpson_) was highest in *flower & berry* sites, where we also found the highest nestedness component (β_JNE_), probably due to hemiedaphic and euedaphic species being more similar in *flower & berry* sites.

Additionally, we found differences between garden land-use types in soil fauna disturbance indices. The collembolan ecomorphological index and the earthworm anecic to endogeic ratio were lowest in *vegetable* sites (Table S2). The acari to collembola ratio was lowest in *grass* sites and the fungal to bacterial ratio was highest in *vegetable* sites.

## Discussion

Worldwide, there is a growing interest of city administrations in the socio-economic and ecological benefits of urban gardens^6,11,12,17,23,29,59^. We investigated impacts of garden management practices on aboveground and belowground diversity and interlinked soil functions. The SEM (Fig. 2, Table 3) revealed direct effects on soil multifunctionality and indirect effects mediated by soil fauna. Overall, our results showed that the largest effects on soil multifunctionality were caused by specific soil characteristics. Soils showing high biological soil quality indices such as organic and microbial carbon and bacteria increased the potential for soil multifunctionality. This probably originates from organic gardening practices such as the application of compost, due to the correlation with increased potassium loads and with decreased bulk density values (Table 3, Table 1), also influencing soil quality^22^. The second strongest effect on soil multifunctionality was caused by the cultivation of vegetables and legumes in *annual vegetable* sites (hypothesis (i), Fig. 1 arrows 1a), probably due to the frequent soil disturbance and the unprotected open soils in comparison to *perennial grass* sites. The cultivation of flowers and berries increased plant diversity (Fig. 1 arrows 1b), but decreased soil fauna biomass compared to *grass* sites. Urban gardens with higher plant diversity (hypothesis (ii), Fig. 1 arrows 3) increased soil multifunctionality directly, and indirectly through increasing fauna diversity and thus fauna biomass. The general pattern of enhanced soil multifunctionality with increased plant diversity is in line with results found in other ecosystems such as croplands, shrublands, grasslands, and forests, where plant diversity increased ES such as pollination, C storage, pest control, and productivity^60,61^. Contrary to our expectations, we found no significant direct effect of management intensity on soil multifunctionality, but more intensively managed sites decreased plant diversity and fauna biomass. A similar relationship of management intensity and decreased diversity has been observed in urban lawns. Lerman *et al*.^62^ showed that mowing only every three weeks instead of every week increased the numbers of flowers by 2.5 times and thus the abundance and diversity of bee populations. Although Tresch *et al*. showed that aboveground^63^ and belowground^22^ organic matter decomposition increased with urbanisation, there was no significant effect of urbanisation (hypothesis (v), Fig. 1 arrow 5), on soil multifunctionality.

The structure of earthworm and springtail communities were influenced by plant ecological indicators (Fig. 3, Table 3), representing the living conditions of plants. Interestingly, springtails were more affected by plant ecological indicators than earthworms, highlighting the dominant influence of plants on springtails^50^. As expected, we found a lower alpha diversity of earthworms and springtails in *vegetable* sites, likely due to the high soil disturbance. Beta diversity was constantly high in both soil fauna and plant communities, driven by high turnover and low nestedness components. The plant community composition was shaped by the high species turnover between the garden sites, with highest dissimilarities for *flower & berry* sites. As expected, these significant differences originated most likely from planting and other garden management practices leading to site specific community compositions. The beta diversity values for both fauna communities were lowest in *vegetable* sites and peaked for earthworms in *grass* sites and for springtails in *flower & berry* sites, reflecting the different ecological strategies of earthworms and springtails.

Earthworms are important indicators for soil functioning^51,52^. Functional groups of earthworms have been used to detect impacts of cultivation in different soils such as pastures, orchards or forest soils, while the ratio of anecic to endogeic species was used as an indicator of contaminated soils^64^, or soil disturbance^65^. In the frequently disturbed *vegetable* sites we found the lowest values for the earthworm anecic to endogeic ratio and the collembolan ecomorphological index, indicating a decreased soil biological quality due to soil disturbance^48^. Other studies have reported the highest value of the collembolan ecomorphological index in urban vegetable gardens and forest sites^48^. However, the highest value found in forest sites (2.3) was still considerably lower than the average we found in urban gardens of Zurich (5.8 ± 0.1), with a high number of euedaphic springtails such as *P*. *pulvinata*, *F*. *quadriculata*, and *I*. *minor*. Springtail abundance often increases from agricultural to forest sites^66^. Here they increased form *vegetable* to *grass* sites by a factor of 4.3. Besides soil disturbance, the increased abundance in grass sites could be explained by the higher plant cover of the perennially vegetated sites^66^ and because grass strips offer a variety of microhabitats for soil mesofauna species^48^. In contrast, Joimel *et al*.^48^ found higher mean densities in vegetable beds than forest or grassland sites, underlining the quality of urban gardens for soil fauna biodiversity and soil quality. In addition, the increased organic matter content in urban garden soils^3^ can be an important factor for the high soil fauna diversity, since the input of organic matter in garden soils can be higher than in agricultural fields^67^. Moreover, high management intensity is known to decrease soil mesofauna diversity^68^. We found a lower acari to collembola ratio in *grass* compared to *flower & berry* sites, which is in line with the dominance of acari in frequently disturbed arable or vineyard soils^48^. Additionally, we found a higher springtail biomass for *grass* sites, while earthworm biomass was at a similar and comparably high level in all urban garden land-use types. For instance, earthworm abundance (227.4 ± 15.5) was considerably higher than mean reference values for biological soil quality indicators found in grass or cropland soils^69^. Referring to all microorganisms^68^, C_mic_ peaked for grass sites, while the mean value (780.9 ± 21.3) was higher than in cropland soils (341 mg kg^−1^), but lower than in grassland soils (1249 mg kg^−1^) found in Belgium^69^ or Switzerland^70^ (2077 mg kg^−1^). This pattern of C_mic_ reflected management practices such as fertilisation or tillage^68^. Additionally, the composition of soil microorganism communities is an important driver for soil functioning^35^. For instance, a shift in fungal composition or activity can increase carbon uptake and nutrient cycling^35^. Both soil disturbance^71^ or lower plant diversity^72^ can result in decreased fungal to bacterial ratios. While increasing fungal to bacterial ratios can be expected from desert to temperate grassland and forest soils, assuming that grassland soils are more bacteria dominated than forest soils^26^. Here, we found an increased fungal to bacterial ratio for *vegetable* sites, due to the increased fungal and decreased bacterial gene copy numbers in those sites (cf. Table S9, Fig. S13). This might be related to the input of compost on the *vegetable* sites or the increased plant diversity compared to *grass* sites.

The intuitive and rather simple concept of multifunctionality^24^ and its reduction to one single metric, such as the averaging approach^37^, needs to be examined critically. For example, the functions and methods to measure them must be carefully selected^42^. The strength of the biodiversity ecosystem multifunctionality relationships depends on the number of included functions, which was generally stronger when more functions were considered^73^. Another point is that the aggregation of multiple functions into one single metric can obscure information about potentially contrasting single functions^74^. The highest correlations among the soil functions (cf. Fig. S14) were found between C_min_ and N_min_ (r = 0.45, p =< 0.001), both used to calculate soil nutrient supply, and between C_min_ and WHC (r = 0.43, p =< 0.001). All other correlations (r < 0.27) claimed a certain independence of the selected soil functions. The moderately positive correlation of all components to soil multifunctionality is required, because negative correlations among functions can be a limitation for multifunctionality assessments^73^. However, this multifunctionality assessment framework could also be used in future studies to assess the impact of managed urban green spaces on nature’s contributions to people in cities.

With this city-wide assessment of the effect of urban gardening practices on aboveground and belowground diversity of plants and soil fauna, we demonstrated the potential impacts of gardeners’ decisions on the quality and functioning of the soil and implications on the biodiversity of a city. In conclusion, our study suggests that a higher plant diversity can directly or indirectly increase soil multifunctionality by enhancing soil fauna diversity and biomass. In a previous study, intensive garden management decreased soil quality indices^22^. Here we demonstrated that a high garden management intensity indeed also declined plant diversity and soil fauna biomass, with negative impacts on soil multifunctionality. In addition, we analysed drivers shaping soil fauna community structure of earthworm and springtail species. We showed that both were affected by plant ecological indicators, soil characteristics, and management practices such as the frequency of soil disturbance or applying water. We conclude that increasing plant diversity together with soil protective management practices have the potential to increase soil functions as well as foster biodiversity, and to create more biophilic^59^ urban gardens, supporting human well-being and the ecological value of urban green spaces. Even though soil is a key resource in cities, it has not been integrated in most urban green space plans^75^, thus we recommend that urban gardens including ecological management practices should be integrated in future green city strategies.

## Methods

### Study design and gradients

This study took place in 85 urban gardens of the city of Zurich, Switzerland (Fig. S1). We selected gardens based on three independent criteria^22,76^: (i) the type of garden (domestic N = 43 vs. allotment; N = 42 Fig. S2), (ii) the management intensity (such as intensively managed vegetable or flower beds or extensively managed meadows), and (iii) the degree of urbanisation, ranging from densely built-up to peripheral areas within the city boundaries. In each garden two sampling plots (2 m × 2 m) with different land-use management were selected (Table S12), belonging to one of the following three categories: annual vegetable beds (*vegetables*; N = 47), perennial flowers and berries (*flowers & berries*; N = 52) or perennial lawn and meadows (*grass*; N = 71), reflecting the most dominant garden land-use types in Zurich and in many other cities.

Garden management practices were assessed using a questionnaire with 26 management questions, specific for each land-use type, ranging from the frequency of lawn cutting to fertiliser application or weeding (Table S3). Garden management intensity was assessed as the sum of 26 management questions. In addition, five common management practices (disturbance, fertiliser, pesticides, water, weeding; Table 2) were used in the community composition analysis. Urban warming was used as a proxy for urbanisation due to the correlation with the amount of built-up and paved area for different radii (30–500 m) around the gardens^63^. It has been assessed as the deviation in local mean air temperatures at night near the surface based on a local climate model^77^, showing temperatures increased of up to 5 °C for urbanised gardens.

### Aboveground diversity

Plant diversity was assessed by a floristic inventory^78^ of cultivated and spontaneously growing plants on each sampling plot (N = 170). Based on this inventory of 600 plant species, we calculated plant alpha diversity as the total number of plant species per sampling plot and plant beta diversity as the mean of the pairwise Jaccard dissimilarity comparisons between each focal plot and all other plots^79^. Additionally, we used a six-point ordinal scale^78^ to calculate community weighted mean values of seven plant ecological indicator values^80^ (Table 2), reflecting the plant environmental requirements^81^.

### Belowground diversity

Earthworms were collected in a smaller subplot of 0.3 m × 0.3 m within the 2 m × 2 m sampling plots by a combined hand sorting and mustard extraction method^22^. Earthworms were stored in 70% ethanol^58^, identified to the species level, and classified into three ecological categories (Table S1): epigeic species (living in the litter layer, with little burrowing activity), endogeic species (living in the soil, with horizontal burrows) and anecic species (living in large and deep vertical burrows).

Springtails and mites were sampled with six undisturbed soil cores (5 cm diameter, 8 cm length, Eijkelkamp, NL) randomly taken in the 2 m × 2 m sampling plots^63^. Springtails were identified to the species level including life forms according to ecological and functional traits (Table S1): epedaphic species (living in the upper litter layer), hemiedaphic species (living at the interface between litter and soil) and euedaphic species (soil-dwelling species).

We defined soil fauna diversity as the average proportional species richness across soil macrofauna (earthworms) and mesofauna (springtails) species following Allan *et al*.^82^. Soil fauna beta diversity was calculated as the average proportional species beta diversity of earthworms and springtails, while the individual measures of beta diversity per soil fauna group were computed as mean pairwise Jaccard dissimilarities, similarly to the plant beta diversity. Soil fauna biomass was calculated as the average proportion of biomass per m^2^ of soil, with measured earthworm biomass [gm^−2^] on an individual basis (including gut contents) and estimated springtail biomass (conversion factor of 5 g for each springtail^83^).

### Soil fauna disturbance indices

The adaptation of soil fauna to management practices was assessed with four soil fauna disturbance indices: the collembolan ecomorphological index^48^, the acari to collembola ratio^84^, the fungal to bacterial ratio^26^, and the earthworm anecic to endogeic ratio^64^.

### Soil characteristics

Soil characteristics were assessed with a combination of three physical, six chemical and four biological soil measurements (Table 1), representing the most commonly used soil quality indicator measurements^85^. The microbial community information of bacterial (16S) and fungal (18S) gene copy numbers were used to calculate the fungal to bacterial ratio. Measurement details can be found in Table S13 and Tresch *et al*.^86^.

### Soil multifunctionality

Similar to other studies^87^ we used the averaging approach^37^ to calculate soil multifunctionality. It calculates the mean value across standardised soil functions for each sampling plot. In total, we used five measurements (Table S11), which are related to important soil functions, for the computation of soil multifunctionality. The three assessed key soil functions are (i) aboveground and belowground litter decomposition, (ii) soil nutrient supply, and (iii) soil water storage and regulation. The soil function litter decomposition aboveground was measured by standardised leaf litter mass loss (*Zea mays* L.) in 4 mm mesh sized litter bags^63^, while belowground litter decomposition was measured by the net mass loss of green tea bags, buried in 8 cm soil depth^22^. The supply of nutrients in the soil was assessed by the mineralisation rates of N (N_min_) and C (C_min_), and the capacity of the soil for water regulation, was measured by the water holding capacity (WHC).

### Data analysis

Soil fauna diversity and biomass were calculated by taking species richness per taxonomic group, applying a standardisation for each taxonomic group scaled to a range from 0 to 1 ($f(x)=({x}_{i}-{x}_{{\min }})$/$({x}_{{\max }}-{x}_{{\min }})$) and then averaging the values for each plot^82^. Aboveground and belowground beta diversity were calculated as mean pairwise Jaccard dissimilarities comparing each focal plot to all other sampling plots^79^ using the R package ‘betapart’^88^. Soil multifunctionality was computed by scaling each of the five measurements of soil functions to a range from 0 to 1^87^ and deriving mean values across the standardised soil functions according to the averaging approach^37^. Community weighted means of plant ecological indicators were calculated with the R package ‘FD’^89^.

We fitted a piecewise structural equation model (SEM), with the ‘piecewiseSEM’ package^57^, to infer relative importance of direct and indirect effects of urban gardening, plant diversity, urbanisation and soil characteristics on soil fauna and soil multifunctionality. To address multicollinearity and reduce the amount of variables we applied a PCA for the soil characteristics and used the first four PCA axes, explaining 64.2% (Table 1; Fig. S4) of the variation (Kaiser-Guttman criteria). We used Shipley’s d-separation test to identify missing paths in the SEM and the AICC for model comparison. We used linear mixed effect models (LMEM; lme(nlme;^90^) with the garden as random effect for each SEM component and reported standardised (scaled by mean and variance) path coefficients, as well as marginal R^2^ and conditional R^2^ based on fixed and random effects^57^ (Table 3). Model assumptions were tested (Fig. S11) and potential spatial autocorrelation patterns were calculated with Moran’s I autocorrelation indices and the spatial structure in the model residuals using semivariograms (Fig. S12).

We applied individual LMEM with garden identity as random effect and land-use types as response variables to assess changes in fauna and plant diversity and soil fauna disturbance indices. We checked for normal distribution, autocorrelation, and heteroscedasticity of the model residuals and applied a transformation ($\mathrm{log}(x+1)$) in the cases of: earthworm biomass, anecic to endogeic ratio, acari to collembola ratio and springtail biomass. We reported means and 95% credible intervals of the Bayesian inference posterior distribution based on 10,000 independent simulations^91^. Soil fauna community structure was further analysed using a permutational multivariate analysis of variance (PERMANOVA, 10,000 permutations) with a Hellinger transformed Euclidean distance species matrix of earthworms (EW) and springtails (COL) and a non-metric multidimensional scaling (NMDS) using the ‘vegan’ package^92^. For the NMDS only significant variables from the PERMANOVA were fitted. Data management and statistical analyses are provided as an R project using R 3.4.2 (R Core Team, 2017).

## Supplementary information

Supplementary Material

Supplementary dataset
