# Supplementary material for: Direct and indirect effects of urban gardening on aboveground and belowground diversity influencing soil multifunctionality
Source: Sci Rep. 2019 Jul 5;9:9769. doi: 10.1038/s41598-019-46024-y (PMC6611818; doi:10.1038/s41598-019-46024-y)
Supplement: Supplementary file 1 — Supplementary Material [file 41598_2019_46024_MOESM1_ESM.pdf]

Supplementary Material

Direct and indirect effects of urban gardening on aboveground and belowground diversity influencing soil multifunctionality

Simon Tresch [1,2,3,*], David Frey [2,4], Renée-Claire Le Bayon [3],Paul Mäder [1], Bernhard Stehle [1,5], Andreas Fliessbach [1] and Marco Moretti [2]

[1] Research Institute of Organic Agriculture (FiBL), Department of Soil Sciences, Ackerstrasse 113, 5070 Frick, CH
[2] Swiss Federal Research Institute WSL, Biodiversity and Conservation Biology, Zuercherstrasse 111, 8903 Birmensdorf, CH
[3] University of Neuchâtel, Institute of Biology, Functional Ecology Laboratory, Rue Emile-Argand 11, 2000 Neuchâtel, CH
[4] ETHZ, Department of Environmental System Science, Institute of Terrestrial Ecosystems, Universitaetstrasse 16, 8092 Zurich, CH
[5] University of Konstanz, Department of Biology, Ecology, Universitaetstrasse 10, 78464 Konstanz, DE

1 Supplementary Figures

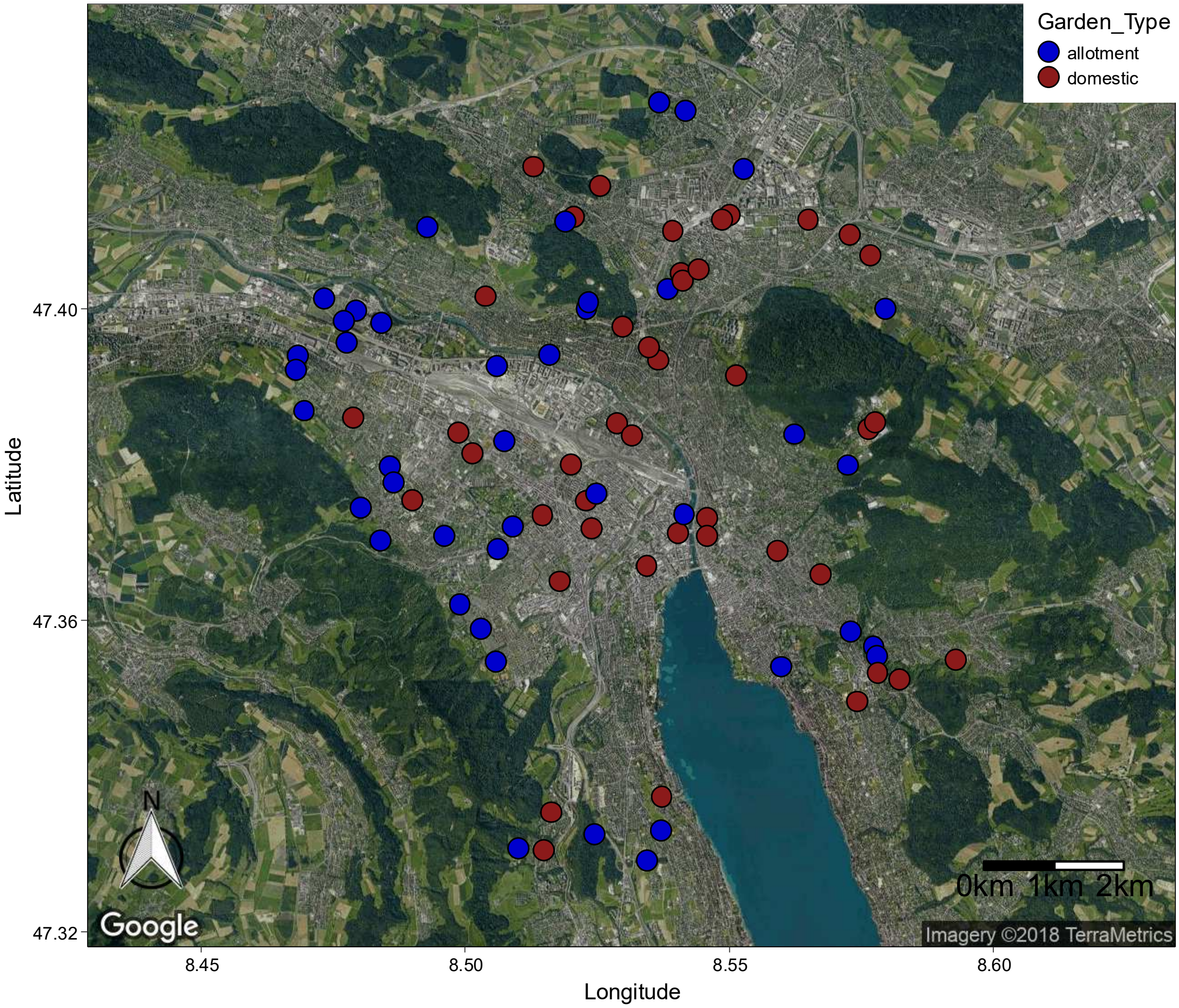

Figure S1. Urban gardens sampled in the city of Zurich. Allotment gardens are displayed in blue (N= 42) and domestic gardens in red (N= 43). Gardens were selected according to the garden type (domestic vs. allotment), the management intensity (extensive vs. intensive garden management), the degree of urbanisation (densely urbanised garden sites vs. peripheral areas). More information on the garden selection can be found in Tresch *et al.*[1] and Frey *et al.*[2]. This figure has been produced using the R package 'ggmap'[3].

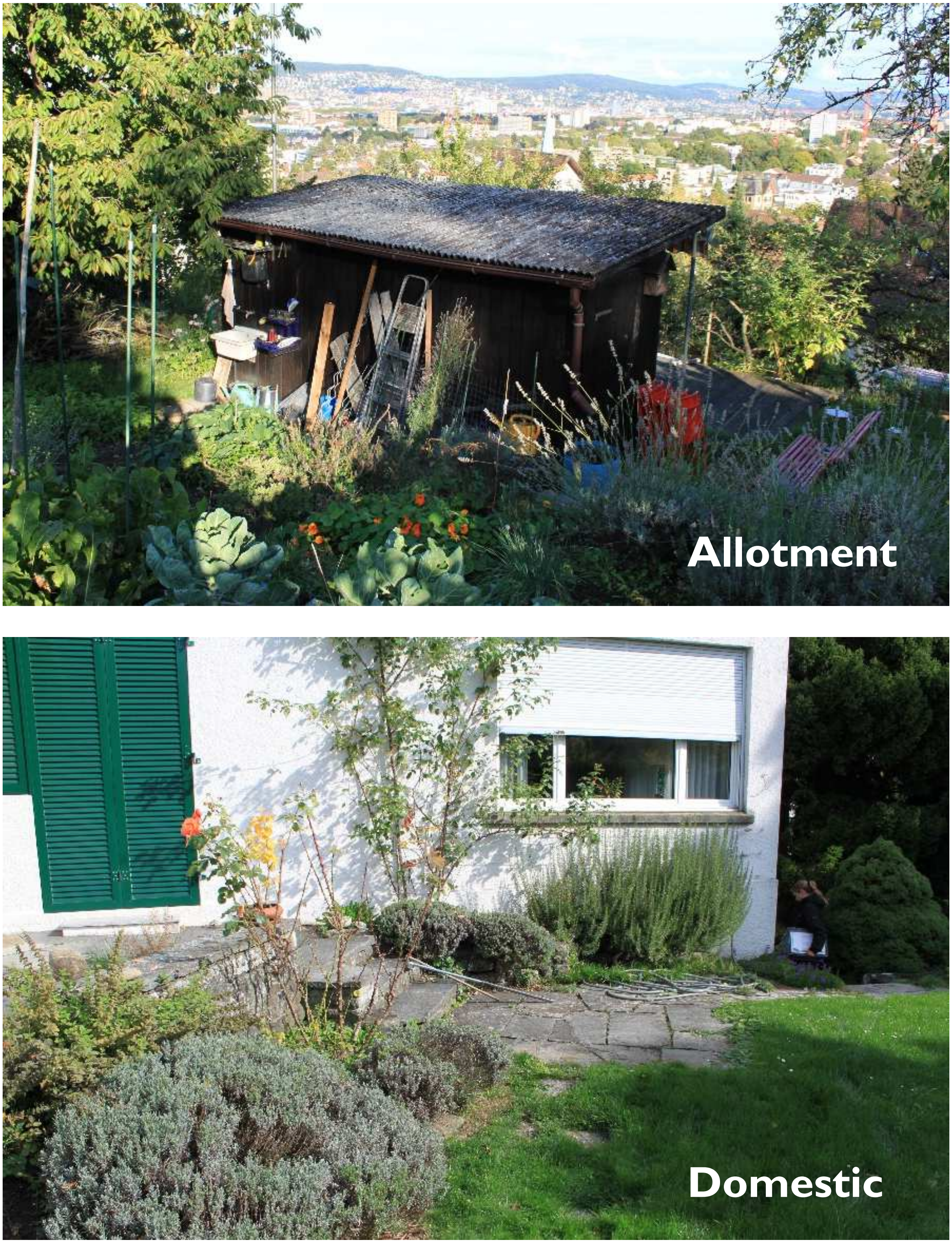

Figure S2. Example of allotment and domestic gardens in the city of Zurich. Within each garden two sampling plots (2 m x 2 m) with different garden land-use management were selected. Each of this sampling plots were later associated with one of the following garden land-use types: annual vegetable beds (vegetables; N= 47), perennial flowers and berries (flowers & berries; N= 52) or perennial lawn and meadows (grass; N= 71). This garden land-use types, rather than the two garden types, have been shown to contain the major differences in soil quality[1] and soil function decomposition[4].

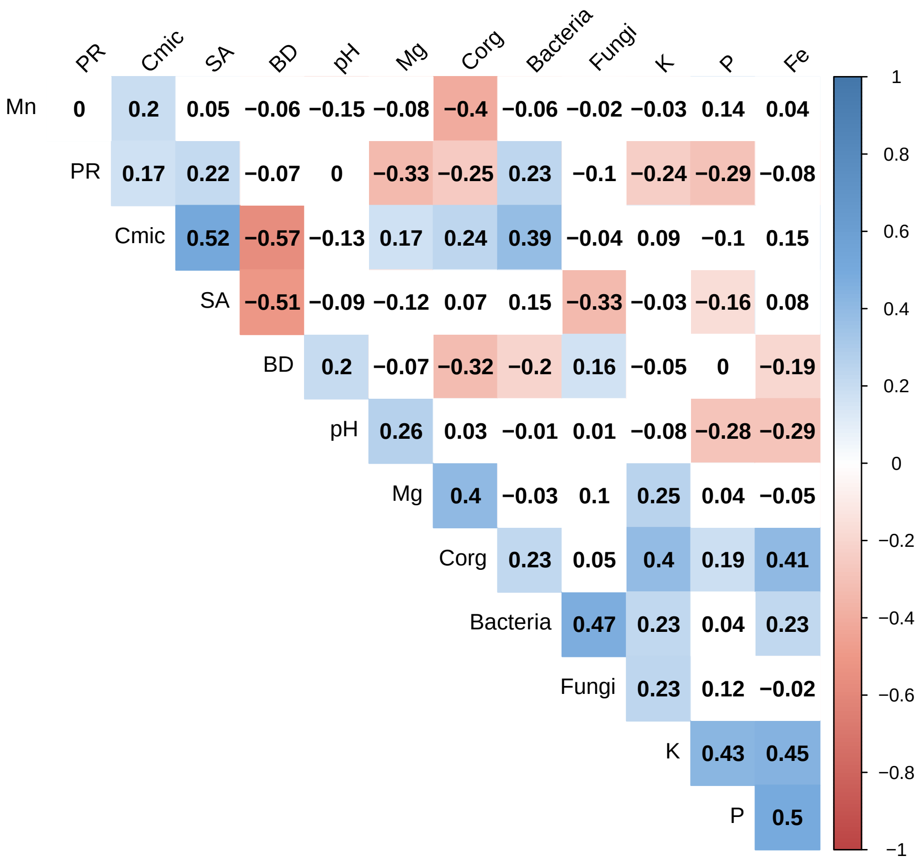

Figure S3. Pearson correlation matrix of selected soil characteristics based on the soil quality assessment of Tresch *et al.*[1]. Only measurements with a very high goodness of fit statistic (p<0.001; see Table S1[1]) for the NMDS ordination, characterising the differences in soil quality between the urban gardens of Zurich have been selected. Additionally, microbial information about gene copy numbers of Bacteria (16S) and Fungi (18S) from Tresch *et al.*[5] has been included in the biological soil characteristics. We dropped Boron because of the high correlation with Potassium (r=0.63) and soil basal respiration because of the correlation with C mineralisation (r=0.98). The overall variation inflation factor[6] is <2.5. SA: Soil stable aggregates, PR: Penetration resistance, BD: Bulk density, Corg: Organic carbon, Cmic: Microbial biomass carbon.

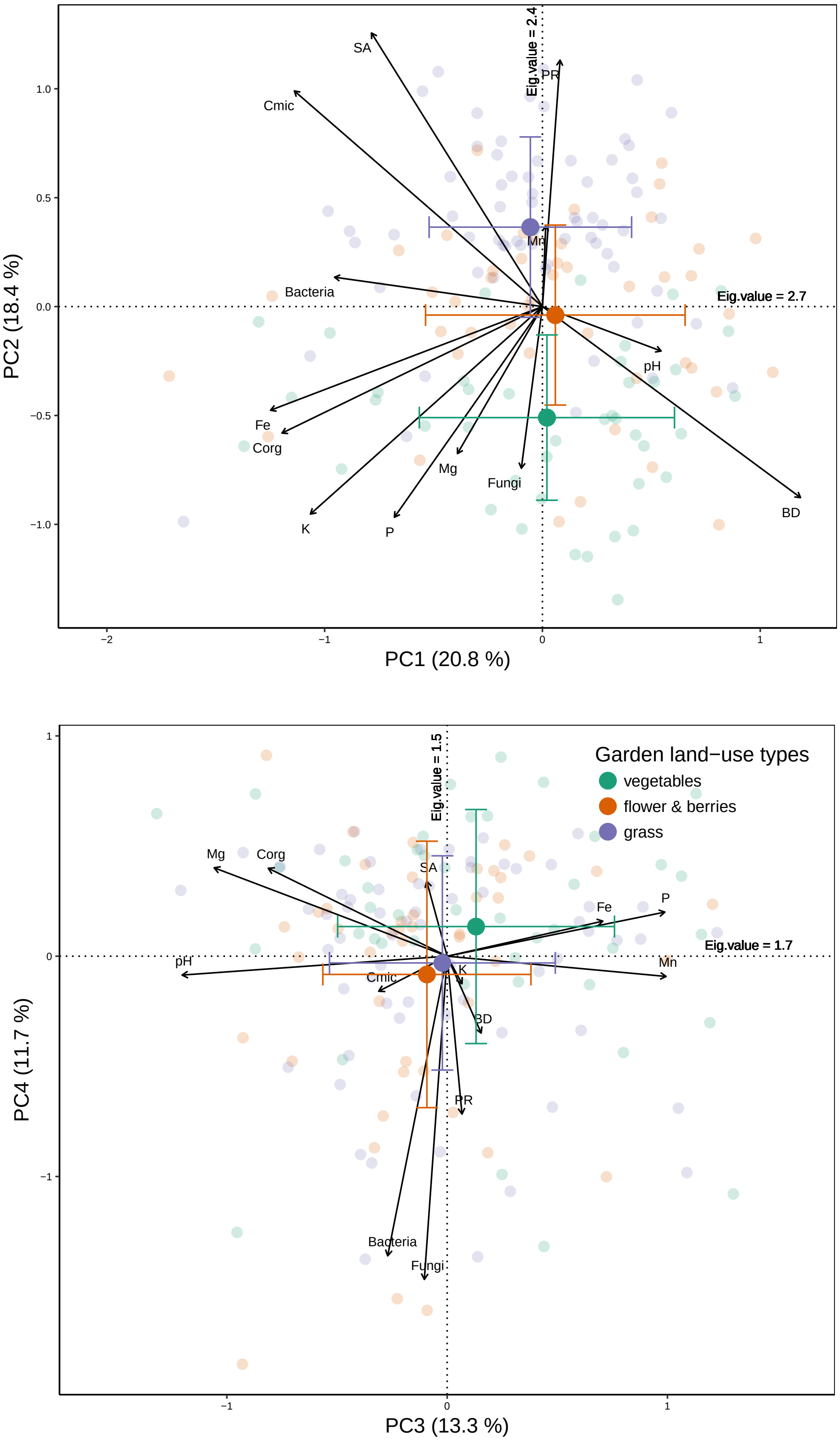

Figure S4. PCA of soil characteristics. First four axes are needed according to the Kaiser-Guttman criteria[6] explaining 64.2 % of the total variation. SA: Soil stable aggregates, PR: Penetration resistance, BD: Bulk density, Corg: Organic carbon, Cmic: Microbial biomass carbon.

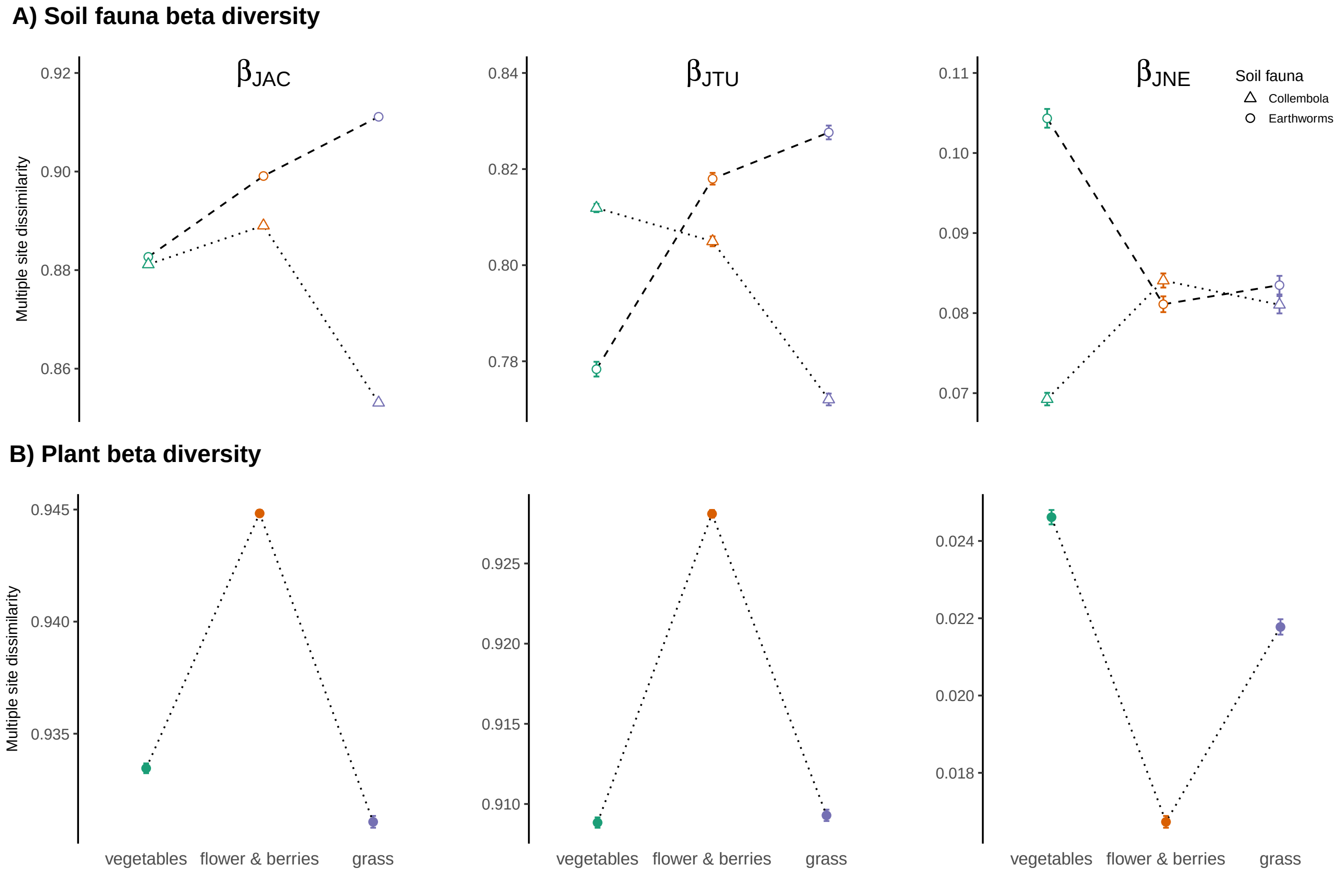

Figure S5. Soil fauna (A) and plant (B) beta diversity components based on species identity, calculated as mean values with 1000 repetitions of 10 plots following Baselga *et al.*[7,8]. β_{JAC}= Total multiple site Jaccard dissimilarity, β_{JTU}= Turnover component, β_{JNE}= Nestedness component. Shapes indicate soil fauna groups and colour the different garden land-use types with error bars as standard errors.

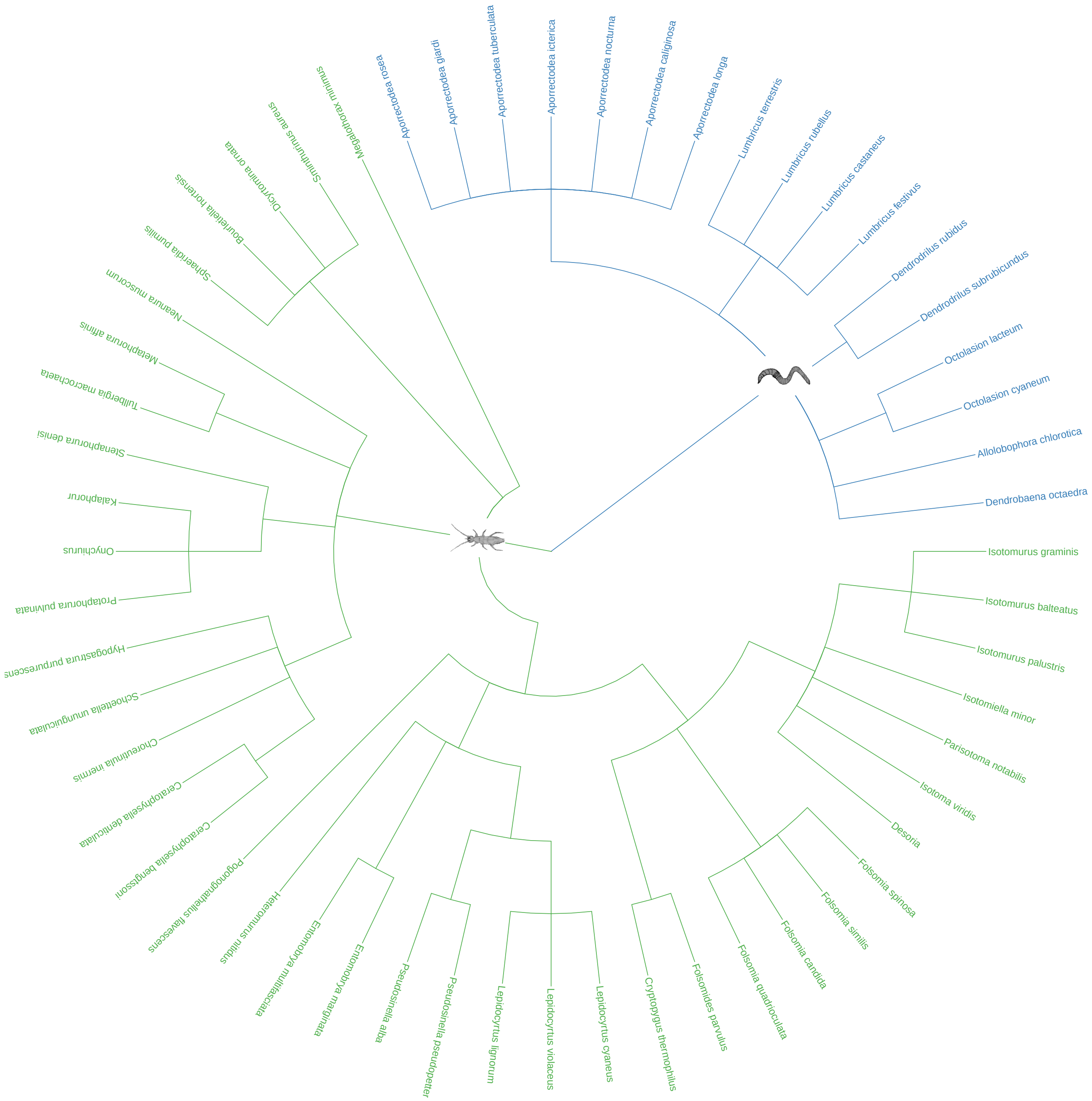

Figure S6. Phylogenetic tree of 18 earthworm and 39 springtail species with branch lengths calculated according to Paradis *et al.*[9] and based on information from the open tree of life project[10].

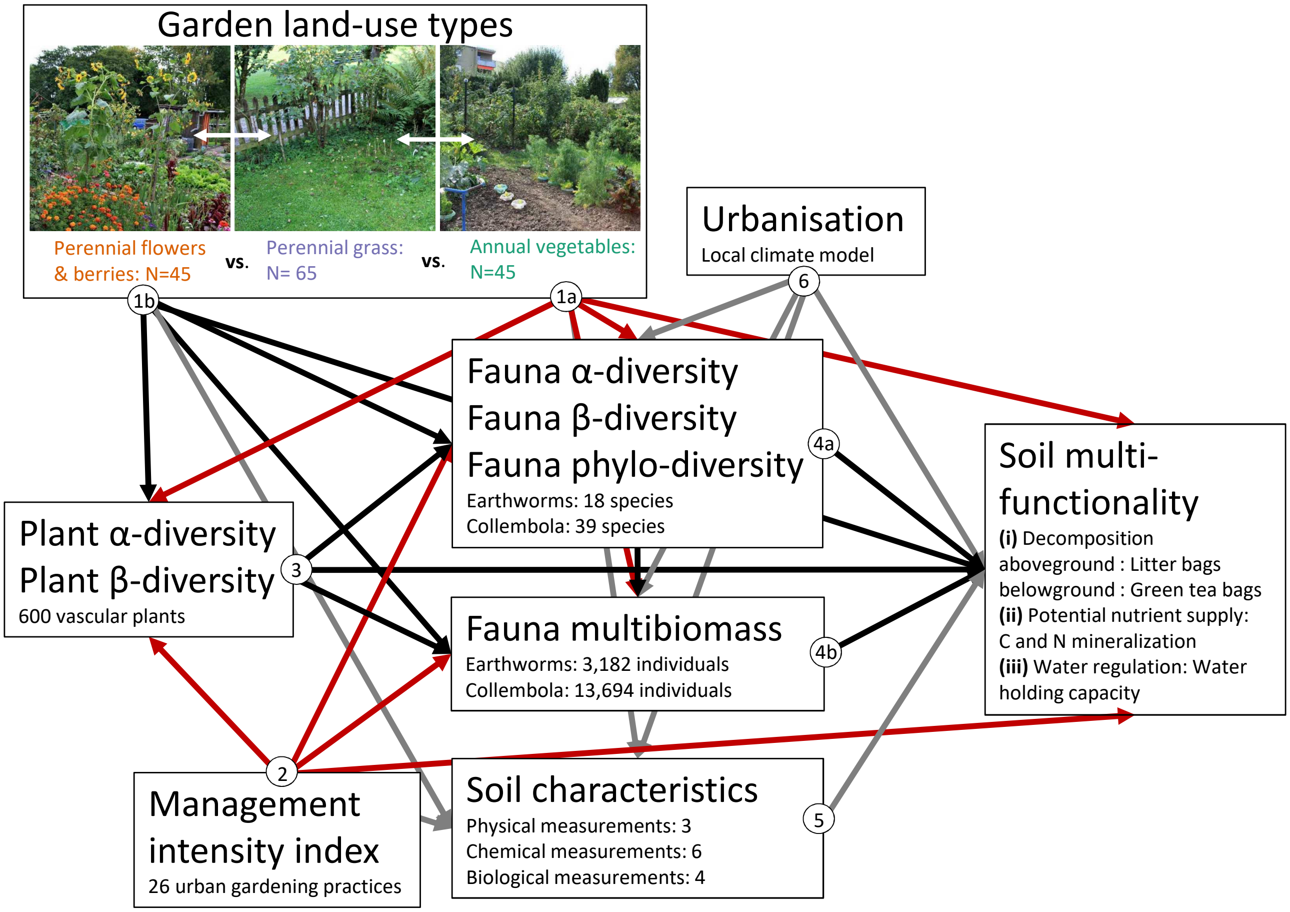

Figure S7. Alternative a priori SEM model investigating the causal relationships between urban gardening and soil multifunctionality. We expected that (1) different garden land-use types (*vegetables*, *flowers & berries*, *grass*) will have an effect on aboveground and belowground α and β-diversity. More specifically, we hypothesised that *vegetables* will have a negative effect on plant and soil fauna α and β-diversities and on soil multifunctionality compared to the other two garden land-use types. (2) Management intensity will negatively affect plant and soil fauna α and β-diversities and soil multifunctionality. (3) Higher plant α and β-diversity will increase soil fauna α and β-diversity and soil multifunctionality. (4) Soil fauna diversity aspects will positively influence soil multifunctionality. Soil characteristics, being affected by management and land-use types and urbanisation will have a direct effect on soil multifunctionality, depending on the measurements. (5) Urbanisation will have an effect on soil fauna and soil multifunctionality. Expected positive relationships are given in black and negative ones in red, grey arrows represent both positive and negative effects.

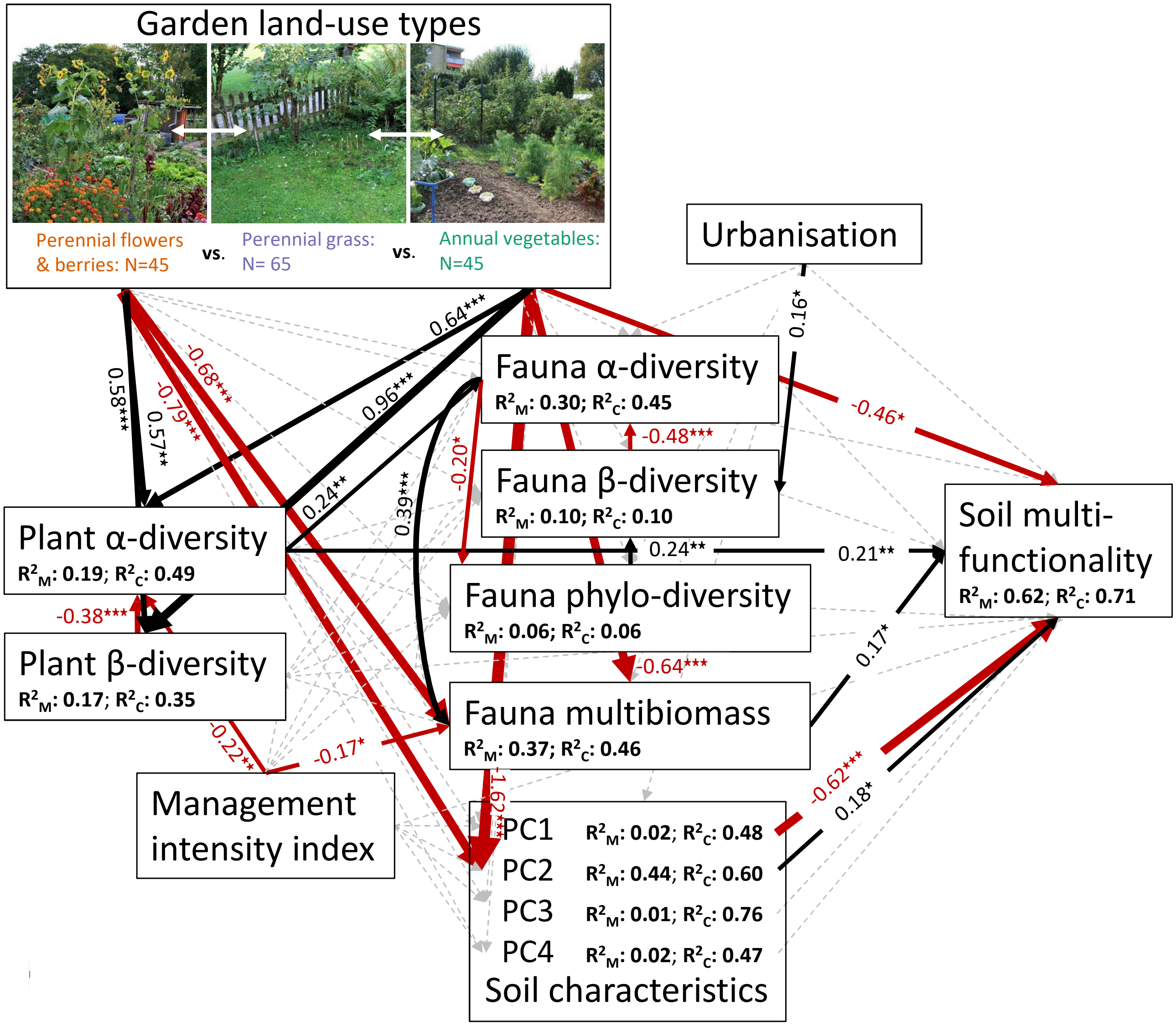

Figure S8. Alternative SEM including plant and soil fauna β-diversity and fauna phylogenetic diversity (AICc=876.8, Fisher's C=45.6, P=0.96). Arrows represent unidirectional relationships among variables. Black arrows denote significantly ($p<0.05$) positive and red arrows significantly negative relationships (Table 7). Dashed grey arrows represent non significant relationships ($p>0.05$). The thickness of paths has been scaled based on the magnitude of the standardised regression coefficient. Conditional R^2's, based on the variance of both the fixed and random effects, as well as marginal R^2's for component models are given in the boxes of response variables. Soil multifunctionality consists of five measurements related to important soil functions.

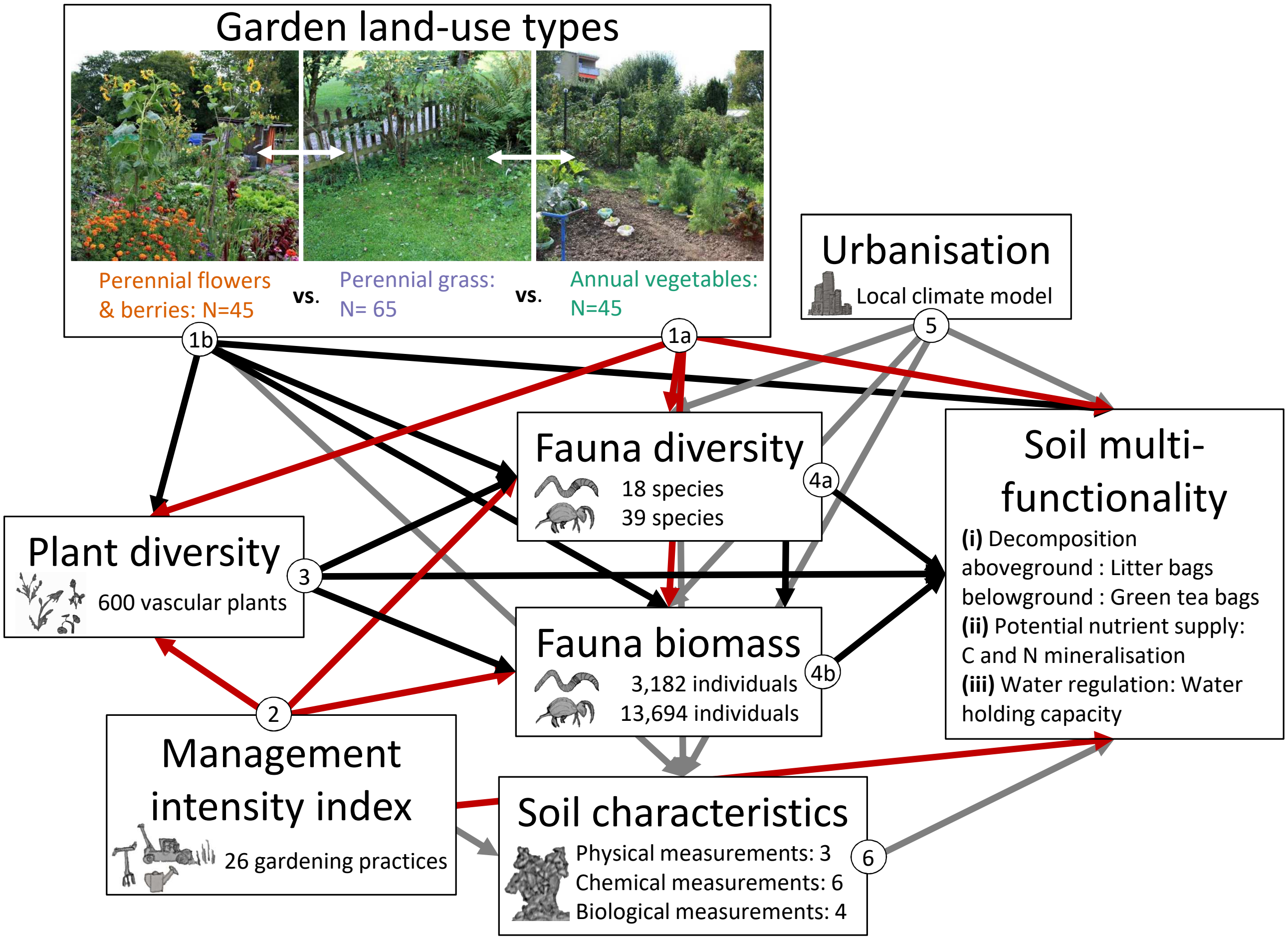

Figure S9. A priori SEM model with hypothesised direct and indirect effects of urban gardening on soil multifunctionality, including soil characteristics (cf. Figure 1). Expected positive relationships are given in black and negative ones in red, grey arrows represent both positive and negative effects. We expected that soil management will negatively affect plant and soil fauna diversity as well as soil multifunctionality (arrows 1 & 2). We hypothesised that higher plant diversity will have a positive effect on soil fauna and soil multifunctionality (arrows 3). We expected a positive effect of soil fauna diversity and biomass on soil multifunctionality (arrows 4). Urbanisation and soil characteristics (arrows 5 & 6) might have a positive or negative effect on soil fauna and soil multifunctionality.

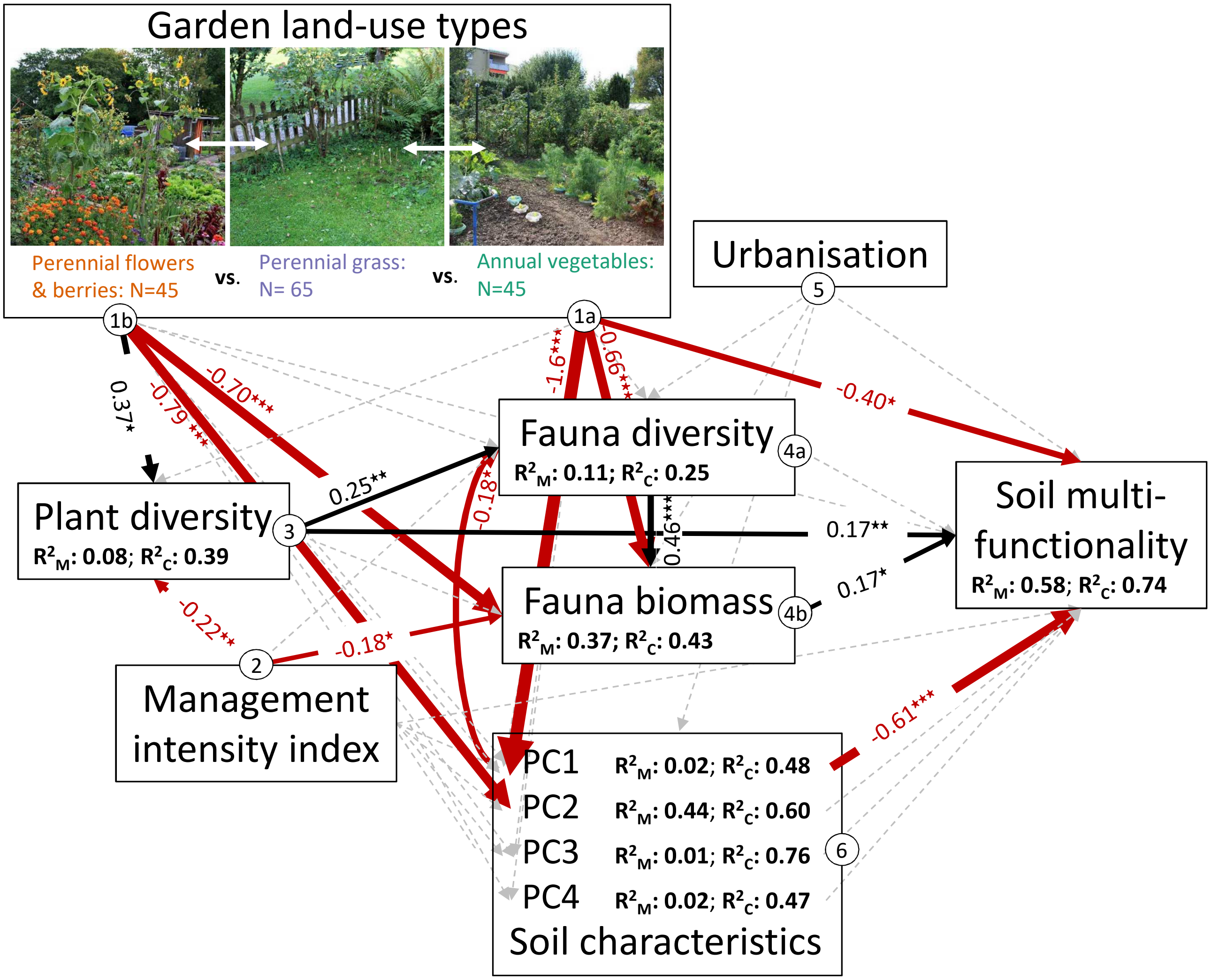

Figure S10. Final most parsimonious SEM (AICc=288.3, Fisher's C=24.3, P=0.93), including soil characteristics (cf. Figure 2). Arrows represent unidirectional relationships among variables. Black arrows denote significantly ($p<0.05$) positive and red arrows significantly negative relationships (Table 4). Dashed grey arrows represent non significant relationships ($p>0.05$). The thickness of paths has been scaled based on the magnitude of the standardised regression coefficient. Conditional R^2s, based on the variance of both the fixed and random effects, as well as marginal R^2s, based on the fixed effect parts for each component models are given in the boxes of the response variables. Soil multifunctionality consists of five measurements related to important soil functions.

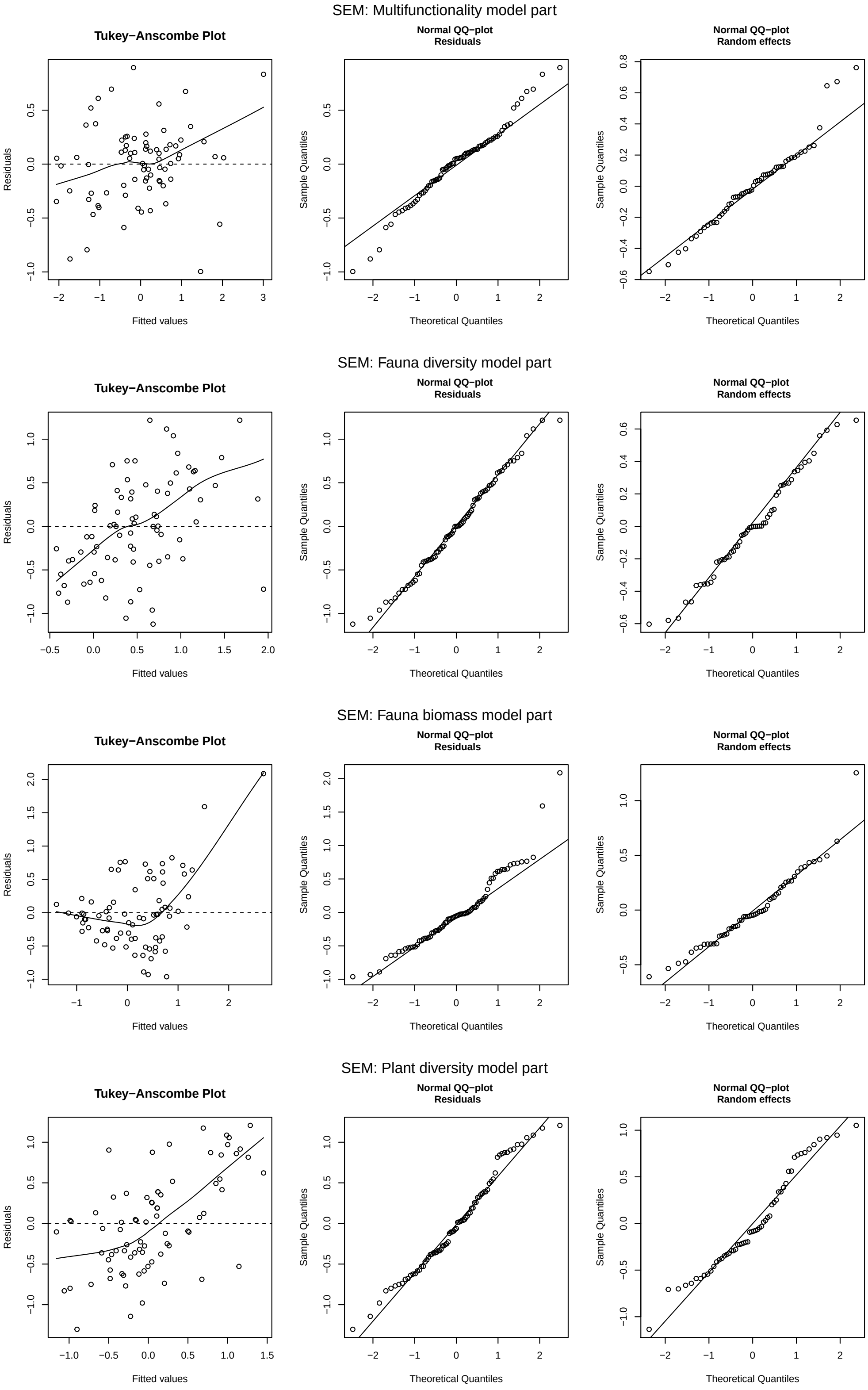

Figure S11. Residual plots for assessing model assumptions of the LMEM used in the SEM (Figure 2). See Table 4 for the complete SEM compositions. Residuals have to be independent and identically distributed, hence they should scatter around zero in the Tukey-Anscombe plots[11]. A few measurements do not fit well to the model as recognisable in the QQ-plots of the residuals, however the majority of the observations seem to fulfil the model assumptions well and since we did not assume a non-linear effect of the assessed variables with the response variables, we accepted the slight contradiction of model assumptions.

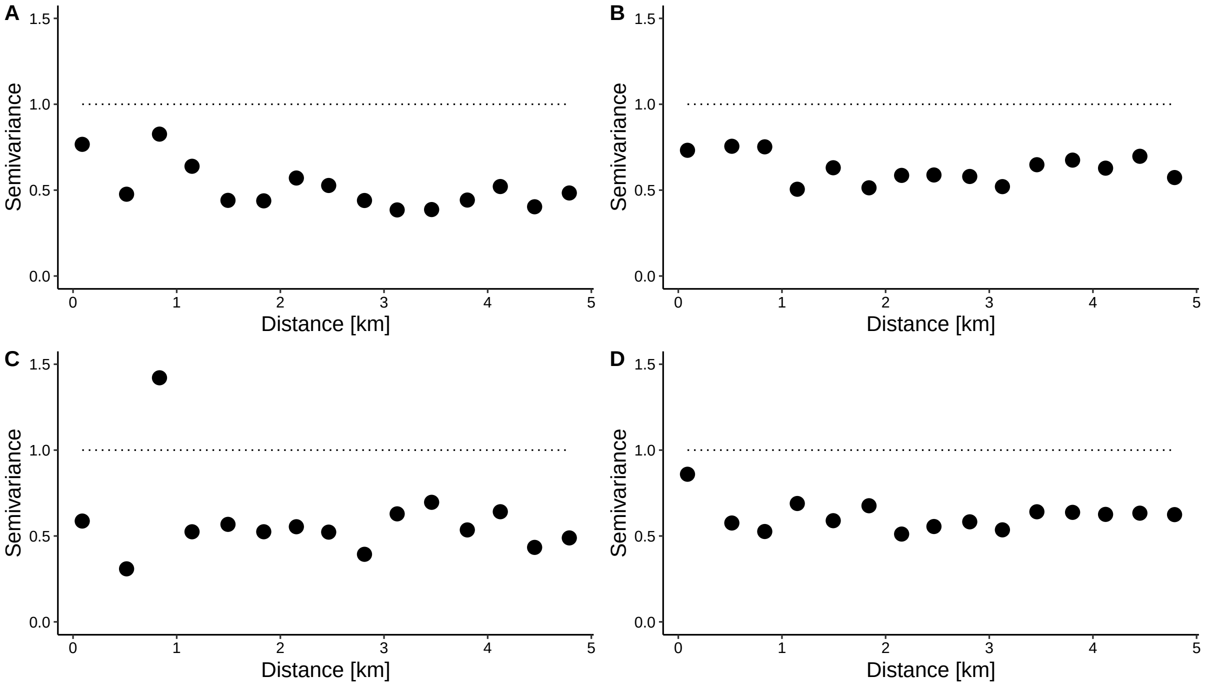

Figure S12. Semivariograms of LMEM residuals from submodels of the SEM (Figure 2): **A)** corresponds to the model part soil multifunctionality, **B)** to the model part fauna diversity, **C)** to the model part fauna biomass, and **D)** to the model part plant diversity. Semivariances (0.5 times the mean squared differences between sites) were computed with the R package 'gstat'[12]. In all plots values are close to 1 and show no clear patterns of spatial autocorrelation, indicating that the residuals are not more similar or dissimilar to each other than expected by chance[11]. Moreover, the calculated Moran's I autocorrelation index[13,14] was not significant for all submodels of the SEM: **A)** p=0.56; observed=-0.01±0.008, expected=-0.013; **B)** p=0.46; observed=-0.02±0.008, expected=-0.013; **C)** p=0.86; observed=-0.01±0.007, expected=-0.013; **D)** p=0.61; observed=-0.01±0.008, expected=-0.013.

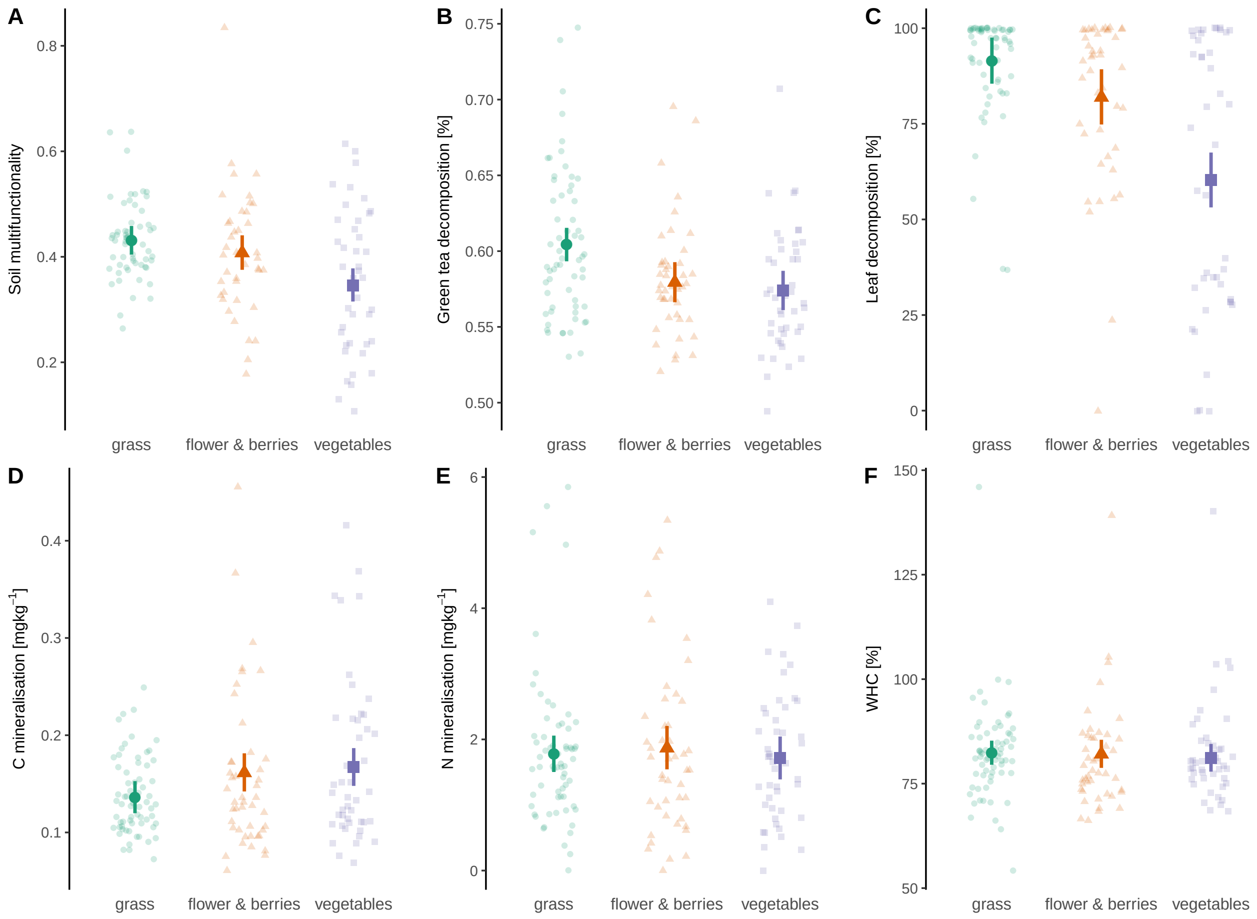

Figure S13. Soil multifunctionality **(A)** and its single components: belowground decomposition of green tea bags **(B)**, aboveground decomposition of leaf litter **(C)**, C mineralisation **(D)**, N mineralisation **(E)** and water holding capacity **(F)**, as a function of garden land-use types. Bold points represent mean values of the simulated Bayesian inference posterior distribution[11] of the LMEM with garden ID as random factor and garden land-use types as fixed effects. Lines indicate 95 % credible intervals. Estimated LMEM coefficients of fixed effects can be found in Table S.5.

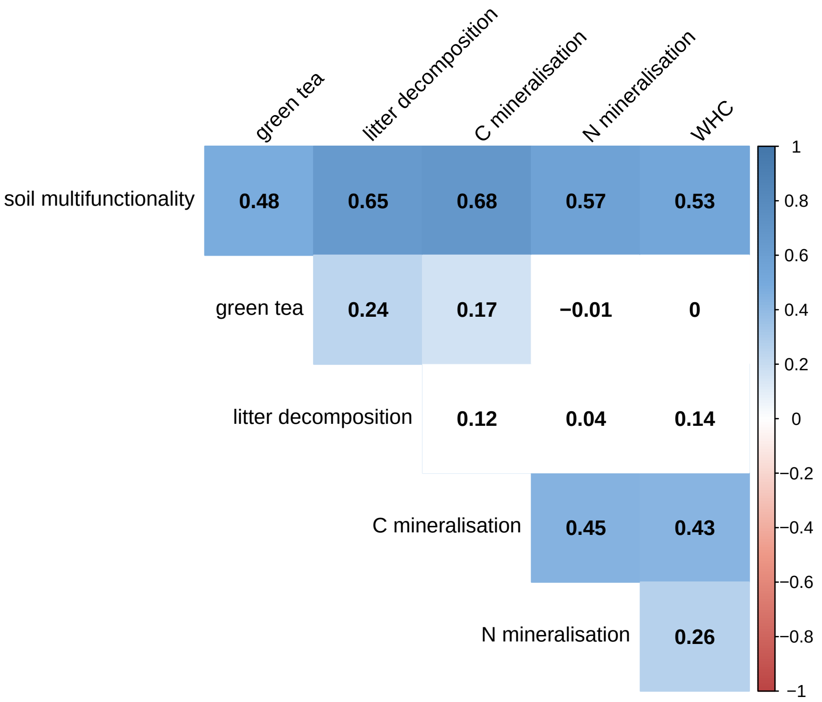

Figure S14. Pearson correlation matrix of soil multifunctionality and its components. WHC: Water holding capacity.

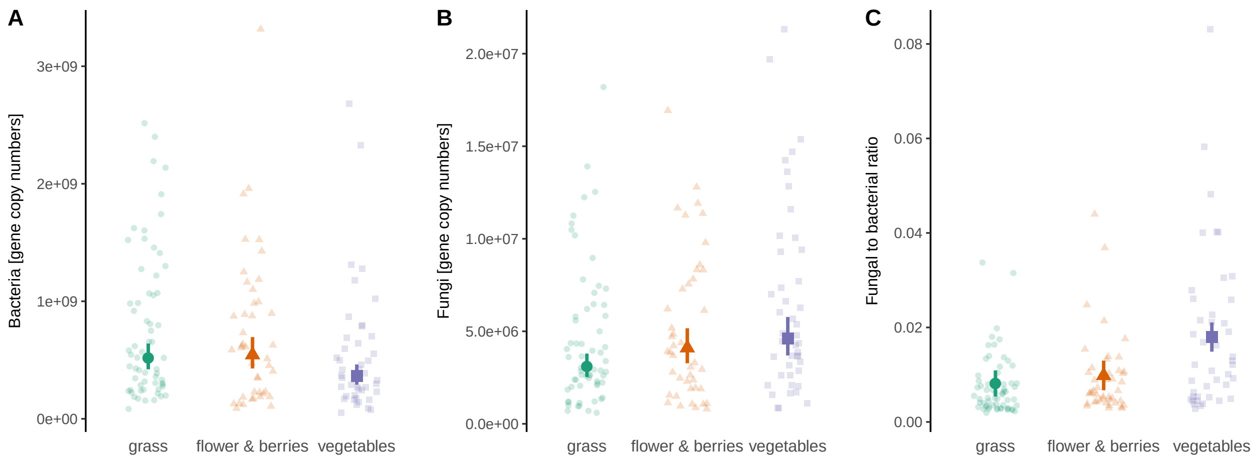

Figure S15. LMEM of bacterial gene copy numbers (**A**), fungal gene copy numbers (**B**) and the fungal to bacterial ratio (**C**), as a function of garden land-use types. Bold points represent mean values of the simulated Bayesian inference posterior distribution[11] of the LMEM with garden ID as random factor and garden land-use types as fixed effects. Lines indicate 95 % credible intervals. Estimated LMEM coefficients of fixed effects can be found in Table S.6.

2 Supplementary Tables

Table S1. Soil fauna species identity and abundance of 39 springtail and 18 earthworm species characterising soil meso- and macrofauna species. Springtails were identified to species level[15–19], while earthworm species identification was done according to Bouché[20] and Sims & Gerard[21]. All earthworm species sampled are already known in Switzerland. The springtail species marked with asterisks, were not listed in Fauna Europaea[22]. The two springtail species with two asterisks are new for Switzerland according to the literature[15,23–31] and expert opinions (Deharveng Luis, Cortet Jérôme, Heiniger Charlène, personal communications, 2018). Earthworm life forms as defined by Bouché[20] and springtail life forms according to their ecological and functional traits as defined by Gisin[24].

Phylum	Class	Order	Family	Genus	Species	Author	Total	Vegetables	Flowers & Berries	Lawn	Life forms
**Springtail species**							13435	2078	2337	9020	
Arthropoda	Collembola	Entomobryomorpha	Isotomidae	*Parisotoma*	*notabilis*	Schaeffer, 1896	3075	292	158	2625	Hemiedaphic
		Poduromorpha	Onychiuridae	*Protaphorura*	*pulvinata*	Gisin, 1954	1810	317	285	1208	Euedaphic
		Symphypleona	Katiannidae	*Sminthurinus*	*aureus*	Lubbock, 1862	1465	130	147	1188	Hemiedaphic
		Entomobryomorpha	Isotomidae	*Folsomia*	*quadrioculata*	Tullberg, 1871	1117	121	351	645	Euedaphic
		Entomobryomorpha	Isotomidae	*Isotomiella*	*minor*	Schaeffer, 1896	817	75	193	549	Euedaphic
		Symphypleona	Bourletiellidae	*Bourletiella*	*hortensis*	Fitch, 1863	801	605	166	30	Epedaphic
		Entomobryomorpha	Isotomidae	*Isotoma*	*viridis*	Bourlet, 1839	685	49	47	589	Epedaphic
		Entomobryomorpha	Entomobryidae	*Pseudosinella*	*petterseni*	Börner, 1901	490	69	55	366	Euedaphic
		Poduromorpha	Hypogastruridae	*Schoettella*	*ununguiculata* *	Tullberg, 1869	423	2	0	421	Hemiedaphic
		Poduromorpha	Hypogastruridae	*Ceratophysella*	*denticulata*	Bagnall, 1941	408	11	303	94	Hemiedaphic
		Entomobryomorpha	Entomobryidae	*Lepidocyrtus*	*lignorum*	Fabricius, 1793	359	48	104	207	Epedaphic
		Entomobryomorpha	Isotomidae	*Folsomia*	*similis*	Bagnall, 1939	343	36	54	253	Euedaphic
		Entomobryomorpha	Entomobryidae	*Pseudosinella*	*alba*	Packard, 1873	303	28	71	204	Euedaphic
		Entomobryomorpha	Entomobryidae	*Cryptopygus*	*thermophilus*	Axelson, 1900	268	152	104	12	Hemiedaphic
		Entomobryomorpha	Isotomidae	*Isotomurus*	*balteatus* **	Reuter, 1876	129	4	24	101	Epedaphic
		Entomobryomorpha	Entomobryidae	*Lepidocyrtus*	*violaceus*	Geoffroy, 1762	121	2	4	115	Epedaphic
		Entomobryomorpha	Isotomidae	*Folsomides*	*parvulus*	Stach, 1920	108	25	34	49	Euedaphic
		Poduromorpha	Tullbergiidae	*Stenaphorura*	*denisi* *	Bagnall, 1935	105	1	33	71	Euedaphic
		Poduromorpha	Tullbergiidae	*Metaphorura*	*affinis*	Börner, 1903	103	10	12	81	Euedaphic
		Entomobryomorpha	Isotomidae	*Folsomia*	*spinosa*	Kseneman, 1936	101	24	55	22	Euedaphic
		Entomobryomorpha	Isotomidae	*Desoria*	*violacea*	Tullberg, 1876	96	4	38	54	Epedaphic
		Entomobryomorpha	Orchesellinae	*Heteromurus*	*nitidus*	Templeton, 1835	93	24	37	32	Hemiedaphic
		Entomobryomorpha	Tomoceridae	*Pogonognathellus*	*flavescens*	Tullberg, 1871	90	6	22	62	Epedaphic
		Poduromorpha	Tullbergiidae	*Mesaphorura*	*macrochaeta*	Rusek, 1976	87	38	25	24	Euedaphic
		Symphypleona	Sminthurididae	*Sphaeridia*	*pumilis*	Krausbauer, 1898	9	0	1	8	Hemiedaphic
		Entomobryomorpha	Entomobryidae	*Lepidocyrtus*	*cyaneus*	Tullberg, 1871	6	0	3	3	Epedaphic
		Entomobryomorpha	Entomobryidae	*Entomobrya*	*multifasciata*	Tullberg, 1871	4	1	3	0	Epedaphic
		Entomobryomorpha	Isotomidae	*Folsomia*	*candida*	Willelm, 1902	3	3	0	0	Hemiedaphic
		Poduromorpha	Onychiuridae	*Kalaphorura*	*burmeisteri* *	Lubock, 1873	3	0	3	0	Euedaphic
		Poduromorpha	Hypogastruridae	*Choreutinula*	*inermis*	Tullberg, 1871	2	1	1	0	Hemiedaphic
		Entomobryomorpha	Isotomidae	*Isotomurus*	*palustris*	Muller, 1776	2	0	1	1	Epedaphic
		Neelipleona	Neelidae	*Megalothorax*	*minimus* *	Willem, 1900	2	0	1	1	Euedaphic
		Poduromorpha	Hypogastruridae	*Ceratophysella*	*bengtssoni*	Agren, 1904	1	0	0	1	Hemiedaphic
		Symphypleona	Dicyrtomidae	*Dicyrtomina*	*ornata*	Nicolet, 1842	1	0	0	1	Epedaphic
		Entomobryomorpha	Entomobryidae	*Entomobrya*	*marginata*	Tullberg, 1871	1	0	0	1	Epedaphic
		Poduromorpha	Hypogastruridae	*Hypogastrura*	*purpurescens* *	Lubock, 1967	1	0	0	1	Hemiedaphic
		Entomobryomorpha	Isotomidae	*Isotomurus*	*graminis* **	Fjellberg, 2007	1	0	1	0	Epedaphic
		Poduromorpha	Neanuridae	*Neanura*	*muscorum*	Templeton, 1835	1	0	0	1	Hemiedaphic
		Poduromorpha	Onychiuridae	*Onychiuroides*	*granulosus* *	Stach, 1930	1	0	1	0	Euedaphic
**Earthworm species**							3169	1253	769	1147	
Annelida	Oligochaeta	-	-	*Endogeic*	*juvenile*	-	1354	567	315	472	Endogeic
		-	-	*Anecic*	*juvenile*	-	609	155	164	290	Anecic
		Opisthopora	Lumbricidae	*Allolobophora*	*chlorotica*	Savigny, 1826	437	282	55	100	Endogeic
		Opisthopora	Lumbricidae	*Aporrectodea*	*caliginosa*	Savigny, 1826	173	78	51	44	Endogeic
		Opisthopora	Lumbricidae	*Lumbricus*	*terrestris*	Linnaeus, 1758	139	24	42	73	Anecic
		Opisthopora	Lumbricidae	*Aporrectodea*	*rosea*	Savigny, 1826	126	36	27	63	Endogeic
		Opisthopora	Lumbricidae	*Aporrectodea*	*longa*	Ude, 1885	90	31	37	22	Anecic
		Opisthopora	Lumbricidae	*Octolasion*	*lacteum*	Örley, 1885	59	33	13	13	Endogeic
		Opisthopora	Lumbricidae	*Aporrectodea*	*nocturna*	Evans, 1946	59	19	19	21	Anecic
		Opisthopora	Lumbricidae	*Allolobophora*	*icterica*	Savigny, 1826	44	5	15	24	Endogeic
		Opisthopora	Lumbricidae	*Aporrectodea*	*tuberculata*	Eisen, 1875	20	8	6	6	Endogeic
		Opisthopora	Lumbricidae	*Aporrectodea*	*ripicola*	Bouché, 1972	20	6	11	3	Anecic
		-	-	Epigeic	juvenile	-	14	6	2	6	Epigeic
		Opisthopora	Lumbricidae	*Dendrobaena*	*octaedra*	Savigny, 1826	6	1	1	4	Epigeic
		Opisthopora	Lumbricidae	*Dendrodrilus*	*subrubicundus*	Eisen, 1874	6	1	3	2	Epigeic
		Opisthopora	Lumbricidae	*Lumbricus*	*castaneus*	Savigny, 1826	4	1	3	0	Epigeic
		Opisthopora	Lumbricidae	*Dendrodrilus*	*rubidus*	Savigny, 1826	3	0	3	0	Epigeic
		Opisthopora	Lumbricidae	*Aporrectodea*	*giardi*	Ribaucourt, 1901	2	0	2	0	Anecic
		Opisthopora	Lumbricidae	*Lumbricus*	*festivus*	Savigny, 1826	2	0	0	2	Epigeic
		Opisthopora	Lumbricidae	*Lumbricus*	*rubellus*	Hoffmeister, 1843	1	0	0	1	Epigeic
		Opisthopora	Lumbricidae	*Octolasion*	*cyaneum*	Savigny, 1826	1	0	0	1	Endogeic

Table S2. Diversity indices of plants and soil fauna. Descriptive statistics (median value ± standard error (SE) and coefficient of variation (CV)) and LMEM fixed effects including means and 95% credible intervals (Mean value (2.5 %; 97.5 %)) of the simulated Bayesian inference posterior distribution[11]. Bold numbers indicate significant effects between two study garden land-use types, with credible intervals not crossing zero. Note that for the calculation of earthworm α-diversity indices only adult species were chosen, due to the dominance of anecic and endogeic juveniles (Table 1).

	Median values ± SE								Fixed effect coefficients 50% (2.5%;97.5%)		
	All sites	CV	Vegetables	CV	Flowers & berries	CV	Grass	CV	Flowers & berries vs. vegetables	Grass vs. vegetables	Grass vs. flowers &berries
**Earthworms α-diversity**											
$D_{Simpson}$	2.0±0.1	52	1.85±0.2	49	2.57±0.2	48	2.0±0.2	55	**0.8 (0.12;1.9)**	0.05 (-0.31;0.61)	-0.41 (-0.62;-0.1)
$D_{Simpson}$ Anecic	1.0±0.05	42	1±0.1	45	1.6±0.09	40	1±0.07	41	0.2 (-0.08;0.57)	-0.06 (-0.27;0.21)	-0.22 (-0.39;-0.01)
$D_{Simpson}$ Endogeic	1.47±0.06	45	1.47±0.1	44	1.8±0.11	44	1.19±0.1	48	0.17 (-0.15;0.61)	0.04 (-0.22;0.39)	-0.11 (-0.35;0.21)
$D_{Simpson}$ Epigeic	1.0±0.02	24	1.5±0.11	47	1±0	0	1±0	0	-	-	-
$E_{Simpson}$	0.9±0.01	22	0.735±0.03	29	0.893±0.02	17	0.9±0.02	17	**0.12 (0.05;0.2)**	**0.17 (0.1;0.24)**	0.04 (-0.02;0.1)
$E_{Simpson}$ Anecic	1±0.01	8	1±0.01	9	1±0.01	8	1±0.01	8	-0.17 (-0.37;0.08)	**-0.22 (-0.39;-0.01)**	0.01 (-0.02;0.05)
$E_{Simpson}$ Endogeic	1±0.01	20	0.9±0.03	26	1±0.02	15	1±0.02	18	**0.09 (0.01;0.16)**	**0.09 (0.02;0.16)**	0.01 (-0.06;0.07)
$E_{Simpson}$ Epigeic	-	-	-	-	-	-	-	-	-	-	-
**Springtail α-diversity**											
$D_{Simpson}$	3.6±0.1	35	3.3±0.2	37	3.4±0.2	42	3.8±0.1	28	0.38 (-0.15;0.93)	0.43 (-0.08;0.92)	0.04 (-0.45;0.53)
$D_{Simpson}$ Epedaphic	1.1±0.05	42	1.0±0.04	25	1.0±0.08	42	1.43±0.08	42	0.14 (-0.11;0.41)	**0.48 (0.24;0.72)**	**0.33 (0.09;0.58)**
$D_{Simpson}$ Hemiedaphic	1.5±0.04	33	1.5±0.09	36	1.2±0.08	39	1.543±0.05	27	0.47 (-0.14;1.54)	0.54 (-0.07;1.52)	0.05 (-0.36;0.69)
$D_{Simpson}$ Euedaphic	2.3±0.09	44	2.0±0.18	52	2.7±0.16	40	2.244±0.12	40	0.47 (-0.07;1.32)	0.12 (-0.26;0.69)	-0.24 (-0.49;0.15)
$E_{Simpson}$	0.52±0.01	33	0.51±0.02	30	0.59±0.03	35	0.49±0.02	30	**0.07 (0.01;0.15)**	-0.04 (-0.11;0.03)	**-0.11 (-0.18;-0.05)**
$E_{Simpson}$ Epedaphic	0.9±0.02	24	1.0±0.03	23	1.0±0.03	22	0.83±0.03	26	-0.01 (-0.08;0.08)	-0.06 (-0.13;0.01)	-0.06 (-0.13;0.02)
$E_{Simpson}$ Hemiedaphic	0.83±0.02	25	0.86±0.03	22	0.96±0.03	23	0.762±0.02	27	0.03 (-0.06;0.12)	-0.07 (-0.14;0.01)	**-0.09 (-0.16;-0.02)**
$E_{Simpson}$ Euedaphic	0.69±0.02	29	0.79±0.03	25	0.76±0.03	30	0.601±0.02	27	-0.06 (-0.13;0.02)	**-0.14 (-0.2;-0.08)**	**-0.09 (-0.15;-0.02)**
**Plants α-diversity**											
$D_{Simpson}$ Plants	25±0.76	36	26±1.58	38	28±1.47	34	23±1.01	33	1.69 (-1.64;5.15)	-2.94 (-6;0.02)	**-4.66 (-7.76;-1.66)**
**Soil fauna disturbance indices**											
Collembolan ecomorphological index	5.8±0.1	30	4.4±0.3	39	6.2±0.2	26	5.7±0.2	24	**1.39 (0.76;2.05)**	**0.85 (0.26;1.43)**	-0.55 (-1.13;0.02)
Acari to collembola ratio	1.3±0.4	236	1.2±0.2	80	1.5±1.2	247	0.99±0.4	189	0.19 (-0.03;0.47)	-0.05 (-0.21;0.14)	**-0.73 (-0.92;-0.07)**
Fungal to bacterial ratio	0.007±0.0009	103	0.012±0.003	93	0.007±0.0001	87	0.006±0.0001	82	**-0.008 (-0.012;-0.004)**	**-0.01 (-0.013;-0.007)**	-0.002 (-0.005;0.001)
Earthworm anecic to endogeic ratio	0.58±0.1	145	0.26±0.05	88	0.67±0.2	115	0.69±0.2	143	**0.34 (0.13;0.59)**	**0.40 (0.19;0.64)**	0.05 (-0.10;0.22)
**Soil fauna biomass**											
Fauna biomass	0.16±0.01	57	0.13±0.01	56	0.12±0.02	70	0.20±0.01	46	0.01 (-0.03;0.05)	**0.07 (0.03;0.1)**	**0.06 (0.02;0.09)**
Earthworm biomass [gm^2]	99.4±6.8	73	97.8±13.6	73	112.2±14.9	80	95.0±8.5	64	0.14 (-0.17;0.59)	-0.02 (-0.27;0.31)	-0.14 (-0.36;0.14)
Springtail biomass [gm^2]	23.98±2.7	95	15.7±1.9	64	18.2±3.4	107	46.7±4.7	71	-0.08 (-0.35;0.3)	**1.42 (0.75;2.32)**	**1.63 (0.91;2.6)**
Microbial biomass (C_{mic}) [$mgkg^{-1}$]	780.85±21.3	33	639.76±36.2	35	772.57±37.0	32	821.63±32.2	29	**106.39 (13.5;202.56)**	**204.83 (119.1;287.83)**	**97.78 (11.35;181.47)**

Table S3. Management questions asked of all 85 participating urban gardeners of this study. Management intensity index was calculated as a scaled sum (divided by the number of questions) of all 26 garden management questions on a five level Likert scale. For *grass* sites we considered nine questions: MowGrass, FstCutGrass, FertGrass, WaterGrass, CareGrass, PestGrass, FlowerIslands, Weeds, Leaves. For *flower & berry* sites ten questions: FertForbs, WaterForbs, PestForbs, DiggingForbs, ForkForbs, CutTrees, PestTrees, Leaves, DrySticks, Weeds and for *vegetables* sites eleven questions: FertCrops, WaterCrops, PestCrops, CropRotate, MixCult, Mulch, GreenFert, DiggingCrops, ForkCrops, DrySticks, Weeds. Furthermore, the following five individual management practices were used: (i) Disturbance: combined management answers (yes/no) of major soil disturbances (”DiggingVeg”, ”DiggingFlower”, ”CareGrass”); (ii) fertiliser: ("FertGrass", "FertVeg", "FertFlower"); (iii) Pesticides: (”PestGrass”,”PestVeg”,”PestFlower”,”PestTrees”,”WeedingHerbicides”); (iv) Water: (”WaterGrass”, ”WaterVeg”, ”WaterFlower”); (v) frequency of weeding ("Weeds"). Higher factor levels indicate higher management intensity. Questions were originally asked in German.

PestGrass
How often do you use pesticides, fungicides or herbicides to protect your lawn?

Never (1)
Less than once per year (2)
1 to 3 times per year (3)
4 to 10 times per year (4)
More than 10 times per year (5)

PestFlower
How often do you use pesticides, fungicides or herbicides (without slug pellets) to protect your flowers?
Never (1)
Less than once per year (2)
1 to 3 times per year (3)
4 to 10 times per year (4)
More than 10 times per year (5)

PestVeg
How often do you use pesticides, fungicides or herbicides (without slug pellets) to protect your vegetables?
Never (1)
Less than once per year (2)
1 to 3 times per year (3)
4 to 10 times per year (4)
More than 10 times per year (5)

FertGrass
How often do you use fertilisers for your lawn?
Never (1)
Every 4 to 5 years (2)
Every 2 to 3 years (3)
Once a year (4)
More than once a year (5)

FertVeg
How often do you use fertilisers for your vegetables?
Never (1)
Every 2 to 3 years (2)
Once a year (3)
2 to 3 times per year (4)
More than three times per year (5)

FertFlower
How often do you use fertilisers for your flowers?
Never (1)
Every 2 to 3 years (2)
Once a year (3)
2 to 3 times per year (4)
More than three times per year (5)

Weeds
How often do you remove most of the weeds in your garden?

Never (1)
Rarely (2)
Sometimes (3)
Often (4)
Very often (5)

PestTrees
How often do you use insecticides, fungicides or herbicides to protect your trees and shrubs?
Never (1)
Less than once a year (2)
1 to 3 times per year (3)
4 to 10 times per year (4)
More than 10 times per year (5)

Leaves
How often do you remove most of the leaves in your garden?

Never (1)
Spring (2)
Autumn (3)
Every 2 to 3 weeks (4)
Weekly in autumn (5)

MowGrass
How often do you mow your lawn?

1 to 2 (1)
3 to 4 (2)
5 to 8 (3)
9 to 20 (4)
over 20 (5)

MixCult
Do you follow the principle of mixed cultivation (planting different varieties of vegetables and/or flowers in the same cultivation plot)?
Never (5)
Rarely (4)
Sometimes (3)
Mostly (2)
Always (1)

FlowerIslands
Do you leave islands of flowers when you mow your lawn?

Never (5)
Rarely (4)
Sometimes (3)
Mostly (2)
Always (1)

WaterGrass
How often do you water your lawn?

Never (1)
When dry (2)
once a week (3)
twice a week (4)
More than twice a week (5)

WaterVeg
How often do you water your vegetable beds?
Never (1)
When dry (2)
once a week (3)
twice a week (4)
More than twice a week (5)

WaterFlower
How often do you water your flower beds?

Never (1)
When dry (2)
once a week (3)
twice a week (4)
More than twice a week (5)

CareGrass
How often do you scarify your lawn (including reseeding)
Never (1)
Every 6 to 10 years (2)
Every 4 to 5 years (3)
Every 2 to 3 years (4)
Annually (5)

DiggingForbs
How often do you till your soil in the flower beds?
Never (1)
Every 3 years or less (2)
Every two years (3)
Once per year (4)
More than once per year (5)

DiggingCrops
How often do you till your soil in the vegetable beds?
Never (1)
Every 3 years or less (2)
Every two years (3)
Once per year (4)
More than once per year (5)

DrySticks
Do you leave withered flowers and sticks during the winter in your garden?
Never (5)
Rarely (4)
Sometimes (3)
Mostly (2)
Always (1)

FstCutGrass
When is the first time point of cutting your lawn?
April (5)
May (4)
Start of June (3)
End of June (2)
After June (1)

Mulch
Do you use organic material (mulch) to cover your vegetable beds?
Never (5)
Rarely (4)
Sometimes (3)
Mostly (2)
Always (1)

CropRotate
Do you consider changing flower beds (crop rotation) for the vegetables grown annually?
Never (5)
Rarely (4)
Sometimes (3)
Mostly (2)
Always (1)

GreenFert
Do you grow plants for green manure?

Never (5)
Rarely (4)
Sometimes (3)
Mostly (2)
Always (1)

WeedingHerbicide
Do you use commercial herbicides?

No (0)
Yes (1)

ForkForbs
How often do you loosen your soil with a fork without turning it around (or milling)?
More than once per year (5)
Once per year (4)
Every 2 years or less (3)
Every 3 years or less (2)
Never (1)

ForkCrops
How often do you loosen your soil with a fork without turning it around (or milling)?
More than once per year (5)
Once per year (4)
Every 2 years or less (3)
Every 3 years or less (2)
Never (1)

CutTrees
How often do you cut most of your forbs and trees?
More than once per year (5)
Once a year (4)
Every 2 years (3)
Every 3 to 5 years(2)
Less than every 5 years (1)

Table S4. NMDS ordination of earthworm and springtail community composition, using goodness of fit measures calculated with 10,000 permutations (envfit{vegan}[32]). Values are ordered according to the pseudo squared correlation coefficient R^2 and printed in bold font if p <0.001.

Earthworm species	R^2	P-value
Allolobophora chlorotica	0.30	< **0.001**
Endogeic juvenile	0.20	< **0.001**
Anecic juvenile	0.19	< **0.001**
Lumbricus terrestris	0.17	< **0.001**
Aporrectodea caliginosa	0.13	< **0.001**
Octolasion lacteum	0.07	0.01
Aporrectodea longa	0.05	0.03
Dendrodrilus rubidus	0.04	0.12
Aporrectodea ripicola	0.03	0.15
Aporrectodea nocturna	0.02	0.17
Lumbricus castaneus	0.02	0.24
Lumbricus festivus	0.02	0.26
Epigeic juvenile	0.02	0.30
Allolobophora icterica	0.01	0.61
Dendrobaena octaedra	0.01	0.43
Dendrodrilus subrubicundus	0.01	0.67
Lumbricus rubellus	0.01	0.49
Octolasion cyaneum	0.01	0.50
Aporrectodea tuberculata	<0.01	0.80
Aporrectodea giardi	<0.01	0.67
Aporrectodea rosea	<0.01	0.83

Springtail species	R^2	P
Bourletiella hortensis	0.29	< **0.001**
Folsomia quadrioculata	0.18	< **0.001**
Parisotoma notabilis	0.14	< **0.001**
Cryptopygus thermophilus	0.13	< **0.001**
Sminthurinus aureus	0.11	< **0.001**
Isotoma viridis	0.10	< **0.001**
Ceratophysella denticulata	0.08	< **0.001**
Pseudosinella alba	0.08	< **0.001**
Metaphorura affinis	0.06	0.01
Pogonognathellus flavescens	0.05	0.02
Schoettella ununguiculata	0.05	0.01
Desoria violacea	0.04	0.05
Entomobrya marginata	0.03	0.09
Entomobrya multifasciata	0.03	0.1
Lepidocyrtus lignorum	0.03	0.09
Lepidocyrtus violaceus	0.03	0.13
Neanura muscorum	0.03	0.08
Heteromurus nitidus	0.02	0.21
Isotomiella minor	0.02	0.19
Pseudosinella petterseni	0.02	0.20
Stenaphorura denisi	0.02	0.22
Choreutinula inermis	0.01	0.66
Folsomia candida	0.01	0.52
Folsomia similis	0.01	0.66
Isotomurus balteatus	0.01	0.62
Kalaphorura burmeisteri	0.01	0.55
Lepidocyrtus cyaneus	0.01	0.48
Megalothorax minimus	0.01	0.34
Mesaphorura macrochaeta	0.01	0.35
Onychiuroides granulosus	0.01	0.55
Protaphorura pulvinata	0.01	0.69
Ceratophysella bengtssoni	<0.01	0.91
Dicyrtomina ornata	<0.01	0.89
Folsomia spinosa	<0.01	0.76
Folsomides parvulus	<0.01	0.81
Hypogastrura purpurescens	<0.01	0.99
Isotomurus graminis	<0.01	0.73
Isotomurus palustris	<0.01	0.95
Sphaeridia pumilis	<0.01	0.77

Table S5. Indices of β-diversity based on species identity of soil fauna (A) and plant (B) communities. All components of β-diversity were calculated by mean values of 1000 repetitions of 10 plots following Baselga *et al.*[7,8]. β_{JAC}= Total multiple site Jaccard dissimilarity, β_{JTU} = Turnover component, β_{JNE}= Nestedness component. Descriptive statistics (median value ± SE) and effects (Mean value (2.5 %; 97.5 %) of the Bayesian posterior distribution (one-way ANOVA) of a linear model with land-use type as fixed effects[11]. Bold numbers indicate significant effects with credible intervals not crossing zero. Note that due to the permutation no random effect could be assigned to the linear model. EW= Earthworm, COL=Collembola, PLA = Plant.

	Median values ± SE				Fixed effect coefficients		
	All sites	Vegetables	Flowers & Berries	Grass	Flowers & berries vs. vegetables	Grass vs. vegetables	Grass vs. flowers & berries
**A) Soil fauna β-diversity components**							
β_{JAC} EW	0.9±0.001	0.883±0.001	0.901±0.001	0.912±0.001	**0.019 (0.018;0.02)**	**0.03 (0.029;0.031)**	**0.011 (0.01;0.012)**
β_{JNE} EW	0.085±0.001	0.099±0.001	0.077±0.001	0.078±0.001	**-0.024 (-0.027;-0.021)**	**-0.022 (-0.025;-0.019)**	**0.002 (-0.001;0.006)**
β_{JTU} EW	0.814±0.001	0.783±0.002	0.824±0.001	0.833±0.001	**0.043 (0.039;0.047)**	**0.052 (0.048;0.055)**	**0.009 (0.005;0.012)**
β_{JAC} COL	0.878±0.001	0.882±0.001	0.889±0.001	0.856±0.001	**0.007 (0.006;0.009)**	**-0.027 (-0.028;-0.026)**	**-0.034 (-0.035;-0.033)**
β_{JNE} COL	0.074±0.001	0.066±0.001	0.081±0.001	0.077±0.001	**0.017 (0.014;0.019)**	**0.014 (0.012;0.017)**	-0.003 (-0.005;0.000)
β_{JTU} COL	0.8±0.001	0.815±0.001	0.805±0.001	0.776±0.001	**-0.01 (-0.012;-0.007)**	**-0.041 (-0.044;-0.038)**	**-0.032 (-0.034;-0.029)**
**B) Plant β-diversity components**							
β_{JAC} PLA	0.937±0.001	0.934±0.001	0.945±0.001	0.932±0.001	**0.011 (0.011;0.012)**	**-0.002 (-0.003;-0.002)**	**-0.014 (-0.015;-0.013)**
β_{JNE} PLA	0.02±0.001	0.024±0.001	0.016±0.001	0.021±0.001	**-0.008 (-0.008;-0.008)**	**-0.003 (-0.003;-0.002)**	**0.005 (0.005;0.006)**
β_{JTU} PLA	0.916±0.001	0.909±0.001	0.929±0.001	0.910±0.001	**0.019 (0.019;0.02)**	0.001 (-0.001;0.001)	**-0.019 (-0.02;-0.018)**

Table S6. Strength of indirect and total pathway estimates of the final SEM (Figure 2, Table 3), calculated by multiplying the standardised coefficients along the path to the response variable and adding the direct pathways[33]. Note that only significant direct and indirect pathways (P<0.05, SEM Table 3) were used.

	Direct pathway estimate	Indirect pathway estimate	**Total estimate**
**Predictors of soil multifunctionality**			
Plant diversity	0.17	$0.25*0.46*0.17 = 0.02$	**0.19**
Annual vegetables	-0.40	$-0.66*0.17 = -0.11$	**-0.51**
PC1	-0.61	$-0.18*0.46*0.17 = -0.01$	**-0.62**
Management intensity	n.s.	$(-0.22*0.17)+(-0.18*0.17)+$ $(-0.22*0.25*0.46*0.17) = -0.07$	**-0.07**
Fauna diversity	n.s	$0.46*0.17 = 0.08$	**0.08**
Perennial flowers & berries	n.s	$(0.37*0.17)+(0.37*0.25*0.46*$ $0.17)+(-0.7*0.17) = -0.05$	**-0.05**
**Predictors of other response variables**			
Management intensity - Fauna biomass	-0.18	$-0.22*0.25*0.46 = -0.03$	**-0.21**
Management intensity - Fauna diversity	n.s.	$-0.22*0.25 = -0.06$	**-0.06**
PC1 - Fauna biomass	n.s.	$(-0.18*0.46) = -0.08$	**-0.08**

Table S7. Alternative SEM (AICc=876.8, Fisher's C= 45.6, P=0.96) including soil fauna and plant β-diversity as well as soil fauna phylogenetic diversity (phyloDiv) indicating direct and indirect effects on soil multifunctionality from garden land-use types, garden management, plant α and β-diversity, soil fauna α and β-diversity, soil characteristics as well as urbanisation. R^2_M is based on fixed effects and R^2_C on fixed and random (garden ID) effects. Soil multifunctionality consisting of five measurements related to important soil functions.

	R^2_C	R^2_M	predictor	estimate	P	
**Soil multifunctionality**	0.71	0.62	Soil PC1	-0.62±0.06	<0.001	***
			Plant α-diversity	0.21±0.06	0.0023	**
			Fauna multibiomass	0.17±0.07	0.02	*
			Vegetables	-0.46±0.20	0.02	*
			Soil PC2	0.18±0.08	0.03	*
			Plant β-diversity	0.13±0.07	0.06	
			Soil PC3	0.11±0.06	0.07	
			Urbanisation	0.11±0.07	0.11	
			Fauna phylo-diversity	0.09±0.06	0.15	
			Management intensity	0.09±0.06	0.19	
			Flowers & berries	-0.11±0.20	0.47	
			Fauna β-diversity	0.05±0.07	0.48	
			Fauna α-diversity	0.03±0.07	0.68	
			Soil PC4	-0.01±0.06	0.91	
**Fauna α-diversity**	0.45	0.30	Fauna β-diversity	-0.48±0.07	<0.001	***
			Plant α-diversity	0.24±0.08	0.0035	**
			Management intensity	0.11±0.08	0.17	
			Flowers & berries	0.09±0.20	0.59	
			Urbanisation	-0.04±0.08	0.61	
			Vegetables	-0.049±0.2	0.79	
			Plant β-diversity	-0.02±0.08	0.81	
**Fauna β-diversity**	0.10	0.10	Fauna phylo-diversity	0.24±0.08	0.0038	**
			Urbanisation	0.16±0.08	0.046	*
			Vegetables	0.40±0.20	0.07	
			Plant α-diversity	-0.06±0.09	0.48	
			Flowers & berries	0.15±0.20	0.48	
			Plant β-diversity	-0.05±0.09	0.63	
			Management intensity	0.001±0.08	0.99	
**Fauna phylo-diversity**	0.06	0.06	Fauna α-diversity	-0.20±0.08	0.023	*
			Flowers & berries	0.35±0.20	0.10	
			Plant β-diversity	-0.08±0.10	0.42	
			Vegetables	0.18±0.20	0.42	
			Plant α-diversity	-0.05±0.09	0.62	
			Management intensity	-0.04±0.09	0.65	
			Urbanisation	-0.02±0.08	0.80	
**Fauna multibiomass**	0.46	0.37	Fauna α-diversity	0.39±0.08	<0.001	***
			Flowers & berries	-0.68±0.20	<0.001	***
			Vegetables	-0.64±0.20	<0.001	***
			Management intensity	-0.17±0.07	0.018	*
			Fauna β-diversity	-0.14±0.08	0.07	
			Plant α-diversity	0.11±0.08	0.17	
			Urbanisation	0.05±0.07	0.50	
			Fauna phylo-diversity	0.03±0.07	0.69	
			Plant β-diversity	0.02±0.08	0.81	
**Plant α-diversity**	0.49	0.19	Plant β-diversity	-0.38±0.08	<0.001	***
			Vegetables	0.64±0.20	<0.001	***
			Flowers & berries	0.58±0.20	<0.001	***
			Management intensity	-0.22±0.08	0.0061	**
**Plant β-diversity**	0.35	0.17	Vegetables	0.96±0.20	<0.001	***
			Flowers & berries	0.57±0.20	0.0013	**
			Management intensity	-0.04±0.08	0.64	
**Soil PC1**	0.48	0.02	Urbanisation	-0.12±0.10	0.24	
			Vegetables	0.17±0.20	0.30	
			Management intensity	-0.05±0.08	0.55	
			Flowers & berries	0.04±0.20	0.81	
**Soil PC2**	0.60	0.44	Vegetables	-1.62±0.10	<0.001	***
			Flowers & berries	-0.79±0.10	<0.001	***
			Management intensity	-0.05±0.06	0.47	
			Urbanisation	0.04±0.07	0.61	
**Soil PC3**	0.76	0.01	Vegetables	0.16±0.10	0.16	
			Management intensity	0.07±0.07	0.32	
			Flowers & berries	-0.02±0.10	0.86	
			Urbanisation	-0.01±0.10	0.90	
**Soil PC4**	0.47	0.02	Vegetables	0.22±0.20	0.18	
			Urbanisation	-0.09±0.10	0.40	
			Flowers & berries	-0.04±0.20	0.83	
			Management intensity	-0.01±0.08	0.93	

Table S8. Estimated LMEM coefficients of soil multifunctionality **(A)** and its single components: belowground decomposition of green tea bags **(B)**, aboveground decomposition of leaf litter **(C)**, C mineralisation **(D)**, N mineralisation **(E)** and water holding capacity **(F)**, as a function of garden land-use types (cf. effect plots Figure S9). Garden ID was set as random effect in all models. Given are the mean, the 2.5% and the 97.5% quantiles of the Bayesian posterior distribution. Bold numbers indicate significant fixed effects, with credible intervals not crossing zero[11].

	Fixed effect coefficients		
	Flowers & berries vs. Grass	Vegetables vs. Grass	Flowers & berries vs. Vegetables
**(A)** Soil multifunctionality	-0.02 (-0.06;0.01)	**-0.08 (-0.12;-0.05)**	**0.06 (0.02;0.10)**
**(B)** Green tea decomposition [%]	**-2.5 (-4.1;-0.8)**	**-3.0 (-4.7;-1.4)**	0.1 (-1.0;2.0)
**(C)** Litter decomposition [%]	**-9.5 (-18.3;-0.7)**	**-31.1 (-39.7;-22.5)**	**21.7 (11.9;31.4)**
**(D)** C mineralisation [$mgkg^{-1}$]	**0.03 (0.01;0.05)**	**0.03 (0.01;0.05)**	-0.01 (-0.03;0.02)
**(E)** N mineralisation [$mgkg^{-1}$]	0.09 (-0.30;0.48)	-0.06 (-0.43;0.32)	0.15 (-0.28;0.58)
**(F)** Water holding capacity [%]	-0.26 (-3.87;3.32)	-1.16 (-4.58;2.27)	0.91 (-3.08;4.88)

Table S9. Estimated LMEM coefficients of Bacteria **(A)**, Fungi **(B)**, and the fungal to bacterial ratio **(C)**, as a function of garden land-use types (cf. effect plots Figure S11). Garden ID was set as random effect in all models. Given are the mean, the 2.5% and the 97.5% quantiles of the Bayesian posterior distribution. Bold numbers indicate significant fixed effects, with credible intervals not crossing zero[11].

	Fixed effect coefficients		
	Flowers & berries vs. Grass	Vegetables vs. Grass	Flowers & berries vs. Vegetables
**(A)** Bacteria [gene copy numbers]	0.05 (-0.21;0.31)	**-0.35 (-0.60;-0.10)**	**0.40 (0.11;0.69)**
**(B)** Fungi [gene copy numbers]	**0.28 (0.05;0.52)**	**0.40 (0.17;0.63)**	-0.12 (-0.38;0.14)
**(C)** Fungal to bacterial ratio	0.002 (-0.002;0.005)	**0.010 (0.007;0.013)**	**-0.008 (-0.010;-0.004)**

Table S10. Soil fauna phylogenetic diversity assessed as phylogenetic species variability (PSV).

Soil fauna phylogenetic diversity, which is usually well correlated with functional diversity in most biodiversity ecosystem functioning studies[34], was assessed as phylogenetic species variability (PSV), representing the mean of the phylogenetic correlations among species, in this case springtail and earthworms, in a community[35]. Phylogenetic trees ('rotl' package[36]) were constructed based on the open tree of life project[10] with branch lengths ('ape' package[9]) to calculate PSV[35].

Table S11. Measurement details of soil functions and properties used to calculate soil multifunctionality.

We used five measurements to calculate soil multifunctionality with the averaging approach[37]. **1.)** The soil function litter decomposition aboveground was measured with litter bags (18 cm x 18 cm; see Finerty *et al.*[38]) with a mesh size of 4 mm on the top and on the bottom a mesh size of 1 mm, in order to prevent smaller pre-decomposed fragments from being lost during the recollection phase. We placed one litter bag on top of the soil layer in each urban garden plot (N=170) for six months (December 2015-May2016), during which most of the leaf litter accumulating in gardens will be decomposed by soil organisms. We only used litter bags with 4 mm mesh size for this calculation and not the ones with 1 mm mesh size on the top of the litter bags, in order to include also macrofauna decomposers in the proxy for aboveground decomposition. Additional leaf litter traits (e.g. C to N ratio or leaf tensile strength) can be found in Tresch *et al.*[4] Table A.1. The litter material (*Zea mays* L.) has been oven dried at 40°C and separated manually into leaf and stem parts before weighing. The starting weight in each litter bag was 2±0.01 g leaf and 2±0.01 g stem material (central leaf vein). Furthermore, only leaf litter has been used, since the mean mass loss (79.6±2.2 %) has been significantly higher compared to the more recalcitrant stems (37.9±20.8 %)[4].

2.) Litter decomposition belowground of mainly soil microfauna[39] was measured by the mass loss of green tea bags in accordance to the tea bag index method by Keuskamp *et al.*[39]. Per garden plot, four replicated tea bags for each tea type (green and rooibos tea) were buried at a depth of 8 cm for 90 days (mid-October until mid-January 2016). The mass loss, expressed as percentage change before and after decomposition was calculated after drying at 60°C and subsequent incineration of the tea bags without the nylon net[5], in order to subtract small soil particles (< 0.25 mm, the size of the tea bag mesh) which possibly entered the tea bags during the phase of decomposition[1]. Only green tea decomposition has been used for the calculation of soil multifunctionality, because of higher mean decomposition rates[39] (59.0±3.7 %) compared to rooibos tea (29.6±2.8 %) found in Tresch *et al.*[1].

3.) Soil nutrient supply has been assessed by the measurements of N_{min} and C_{min}. N_{min} was measured in an extract with 0.01 M $CaCl_2$ (1:4 w/v) following Krauss *et al.*[40].

4.) C_{min} rates were calculated as cumulative values after 4 weeks by incubating 30 g moist soil (40-50 % water holding capacity) at 20°C. CO_2 flux calculations were based on the increase of CO_2 concentration in the head-space over 6 hours, measured once per week for the 4 week time period with a gas chromatograph (7890A, Agilent Technologies, USA) as described in Tresch *et al.*[1] Table S12. The linearity of the enrichment was tested according to Krause *et al.*[41].

5.) The capacity of the soil to store water was measured by the soil water holding capacity (WHC). We measured WHC with a cylinder method, where field moist soil is saturated with water on a sand bath following Schinner *et al.*[42].

Table S12. Urban garden land-use types by garden types. Table A) displays all sampled urban garden plots. Table B) illustrates total number of observations without NA's used for the SEM and other statistical analyses. The discrepancy in observations is due to many reasons, such as missing litter bags on some sites or missing values in laboratory analyses for the measurement of the soil quality indices.

**(A)**	Urban garden land-use types			
	Perennial flowers & berries	Perennial grass	Annual vegetables	**Total**
Allotment garden sites	19	29	36	**84**
Domestic garden sites	33	42	11	**86**
**Total**	**52**	**71**	**47**	**170**

**(B)**	Urban garden land-use types			
	Perennial flowers & berries	Perennial grass	Annual vegetables	**Total**
Allotment garden sites	18	27	34	**79**
Domestic garden sites	27	38	11	**76**
**Total**	**45**	**65**	**45**	**155**

References

1. Tresch, S. *et al.* A Gardener's Influence on Urban Soil Quality. *Front. Environ. Sci.* **6**, DOI: 10.3389/fenvs.2018.00025 (2018).
2. Frey, D. & Moretti, M. A comprehensive dataset on cultivated and spontaneously growing vascular plants in urban gardens. *Data Br.* **in press**, 103982, DOI: 10.1016/j.dib.2019.103982 (2019).
3. Kahle, D. & Wickham, H. ggmap: Spatial Visualization with ggplot2. *R J.* **5**, 144–161 (2013).
4. Tresch, S. *et al.* Litter decomposition driven by soil fauna, plant diversity and soil management in urban gardens. *Sci. Total. Environ.* **658**, 1614–1629, DOI: 10.1016/j.scitotenv.2018.12.235 (2019).
5. Tresch, S. *et al.* Urban Soil Quality Assessment—A Comprehensive Case Study Dataset of Urban Garden Soils. *Front. Environ. Sci.* **6**, 1–5, DOI: 10.3389/fenvs.2018.00136 (2018).
6. Borcard, D., Gillet, F. & Legendre, P. *Numerical Ecology with R* (Springer New York, New York, NY, 2011).
7. Baselga, A. Partitioning the turnover and nestedness components of beta diversity. *Glob. Ecol. Biogeogr.* **19**, 134–143, DOI: 10.1111/j.1466-8238.2009.00490.x (2010).
8. Baselga, A. & Orme, C. D. L. Betapart: An R package for the study of beta diversity. *Methods Ecol. Evol.* **3**, 808–812, DOI: 10.1111/j.2041-210X.2012.00224.x (2012).
9. Paradis, E., Claude, J. & Strimmer, K. APE: Analyses of Phylogenetics and Evolution in R language. *Bioinformatics* **20**, 289–290, DOI: 10.1093/bioinformatics/btg412 (2004).
10. Hinchliff, C. E. *et al.* Synthesis of phylogeny and taxonomy into a comprehensive tree of life. *Proc. Natl. Acad. Sci.* **112**, 12764–12769, DOI: 10.1073/pnas.1423041112 (2015). 1503.03877.
11. Korner-Nievergelt, F. *et al. Bayesian data analysis in ecology using linear models with R, BUGS, and Stan* (Academic Press, 2015).
12. Pebesma, E. J. Multivariable geostatistics in S: the gstat package. *Comput. Geosci.* **30**, 683–691 (2004).
13. Popescu, A.-A., Huber, K. T. & Paradis, E. ape 3.0: New tools for distance-based phylogenetics and evolutionary analysis in R. *Bioinformatics* **28**, 1536–1537 (2012).
14. Paradis, E. Moran's autocorrelation coefficient in comparative methods. Tech. Rep. (2018).
15. Gisin, H. Collembolenfauna Europas. Tech. Rep., Museum d'Histoire Naturelle, Genève (1960).
16. Zimdars, B. & Dunger, W. *Synopses on Palaearctic Collembola: Tullbergiinae.* (Abh. Ber. Naturkundemus, Görlitz, 1994), 68 edn.
17. Fjellberg, A. *The Collembola of Fennoscandia and Denmark, Part I: Poduromorpha* (Brill, 1998).
18. Fjellberg, A. *The Collembola of Fennoscandia and Denmark, Part II: Entomobryomorpha and Symphypleona* (Brill, 2007).
19. Bretfeld, G. *Synopses on Palaearctic Collembola: Symphypleona* (Abh. Ber. Naturkundemus, Görlitz, 1999), 71 edn.
20. Bouché, M. *Lombriciens de France. Ecologie et systématique* (INRA Editions, Paris, 1977), annales de edn.
21. Sims, R. W. & Gerard, B. M. *Earthworms: Notes for the identification of British species* (Linnean Society of London and the Estuarine and Coastal Sciences Association, 1999).
22. Deharveng, L. Fauna Europaea: Collembola (2018).
23. Handschin, E. Die Collembolenfauna des Schweizerischen Nationalparkes. *Neue Denkschriften der Allg. Schweizerischen Gesellschaft fur die gesammten Naturwissenschaften* **60**, 89–174 (1924).
24. Gisin, H. *Ökologie und Lebensgemeinschaften der Collembolen im schweizerischen Exkursionsgebiet Basels*, vol. 50 (1943).
25. Gisin, H. Révision des espèces suisses du genre Bourletiella s. lat. (Collembola). *J. Swiss Entomol. Soc.* **20**, 249–261 (1946).
26. Gisin, H. Etudes écologiques sur les Collemboles épigés. *J. Swiss Entomol. Soc.* **21** (1948).
27. Gisin, H. *Collembolen einiger Waldböden des Fuorngebietes* (Lüdin, Liestal, 1957).
28. Dunger, W., Schulz, H.-J., Zimdars, B. & Hohberg, K. Changes in collembolan species composition in Eastern German mine sites over fifty years of primary succession. *Pedobiologia (Jena).* **48**, 503–517, DOI: https://doi.org/10.1016/j.pedobi.2004.07.005 (2004).

29. Schulz, H. J. *Springschwänze (Collembola) der Alp Flix: erste Ergebnisse*, vol. 114 (Jber. Natf. Ges. Graubünden, 2007).

30. Troxler, C. *Vergleichende bodenbiologische Untersuchungen in Grünland- und Ackerflächen: Mikroarthropoden (Acari, Collembola) als Bioindikatoren und mikrobielle Aktivität, insbesondere Zelluloseabbau.* Ph.D. thesis, Bern (1990).

31. Rusterholz, H.-P., Salamon, J.-A., Ruckli, R. & Baur, B. Effects of the annual invasive plant Impatiens glandulifera on the Collembola and Acari communities in a deciduous forest. *Pedobiologia (Jena).* **57**, 285–291, DOI: 10.1016/j.pedobi.2014.07.001 (2014).

32. Oksanen, J. *et al. vegan: Community Ecology Package* (2017).

33. Lefcheck, J. S. piecewiseSEM: Piecewise structural equation modelling in r for ecology, evolution, and systematics. *Methods Ecol. Evol.* **7**, 573–579, DOI: 10.1111/2041-210X.12512 (2016). 1509.01845.

34. Gessner, M. O. *et al.* Diversity meets decomposition. *Trends Ecol. Evol.* **25**, 372–380, DOI: 10.1016/j.tree.2010.01.010 (2010).

35. Paradis, E. *Analysis of Phylogenetics and Evolution with R* (Springer New York, New York, NY, 2011). arXiv:1011.1669v3.

36. Michonneau, F., Brown, J. W. & Winter, D. J. rotl : an R package to interact with the Open Tree of Life data. *Methods Ecol. Evol.* **7**, 1476–1481, DOI: 10.1111/2041-210X.12593 (2016).

37. Byrnes, J. E. K. *et al.* Investigating the relationship between biodiversity and ecosystem multifunctionality: Challenges and solutions. *Methods Ecol. Evol.* **5**, 111–124, DOI: 10.1111/2041-210X.12143 (2014). arXiv:1011.1669v3.

38. Finerty, G. E. *et al.* Exotic or not, leaf trait dissimilarity modulates the effect of dominant species on mixed litter decomposition. *J. Ecol.* **104**, 1400–1409, DOI: 10.1111/1365-2745.12602 (2016).

39. Keuskamp, J. A., Dingemans, B. J. J., Lehtinen, T., Sarneel, J. M. & Hefting, M. M. Tea Bag Index: A novel approach to collect uniform decomposition data across ecosystems. *Methods Ecol. Evol.* **4**, 1070–1075, DOI: 10.1111/2041-210X.12097 (2013).

40. Krauss, M. *et al.* Tillage system affects fertilizer-induced nitrous oxide emissions. *Biol. Fertil. Soils* **53**, 49–59, DOI: 10.1007/s00374-016-1152-2 (2017).

41. Krause, H.-M. *et al.* Long term farming systems affect soils potential for N 2 O production and reduction processes under denitrifying conditions. *Soil Biol. Biochem.* **114**, 31–41, DOI: 10.1016/j.soilbio.2017.06.025 (2017).

42. Schinner, F., Öhlinger, R., Kandeler, E. & Margesin, R. *Methods in Soil Biology* (Springer Berlin Heidelberg, Berlin, Heidelberg, 1996).
